# Scaffolds and Artificial Augmentations for Tendon Repair

**DOI:** 10.3390/ijms27209072

**Published:** 2026-10-13

**Authors:** Gabriel Kordahji, Dor Yakoby, Ben Kaplan

**Affiliations:** 1 Department of Orthopedic Surgery, Carmel Medical Center, Haifa 3436212, Israel; kordahjigabriel@gmail.com; 2 Bruce Rappaport Faculty of Medicine, Technion-Israel Institute of Technology, Haifa 35254, Israel; dory298@gmail.com; 3 Department of Orthopedic Surgery, Rambam Health Care Campus, Haifa 3109601, Israel

**Keywords:** tendon injuries, biomaterials, scaffolds, stem cells, artificial tendons

## Abstract

Tendon injuries are common musculoskeletal disorders that represent a significant clinical challenge due to the limited regenerative capacity of tendon tissue. Although surgical repair remains the standard treatment for severe tendon injuries, healed tendons rarely regain the structural and mechanical properties of native tissue, often resulting in impaired function and an increased risk of re-injury. Recent advances in tissue engineering have introduced biologically active and biomimetic strategies aimed at enhancing tendon healing and promoting functional regeneration. This narrative review summarizes current knowledge of tendon injuries and highlights emerging tissue engineering strategies for tendon repair and regeneration. Emphasis is placed on the design and application of natural and synthetic polymers, scaffold engineering, and the key biological and mechanical characteristics required for successful tendon regeneration. In addition, recent developments in drug delivery systems, mesenchymal stem cell-based therapies, and artificial tendon technologies are discussed. These emerging approaches offer promising alternatives and adjuncts to conventional treatments, with the potential to improve tendon healing and functional recovery. However, further studies are required to optimize these technologies, establish their long-term safety and efficacy, and facilitate their translation into routine clinical practice.

## 1. Introduction

Tendon ruptures and lacerations are common musculoskeletal injuries that cause significant pain, increased healthcare costs, and substantial disability [1,2]. Management depends on the tendon involved, injury characteristics, and individual patient factors. Surgical repair is commonly indicated for complete traumatic lacerations and selected tendon ruptures, whereas non-operative treatment may provide satisfactory outcomes for selected acute ruptures. Chronic tendinopathy represents a distinct clinical entity and is generally managed initially with non-operative strategies, particularly exercise-based rehabilitation, with adjunctive modalities considered according to the specific tendon and clinical presentation [3,4,5].

Tendon repair has long been a clinical challenge in orthopedic surgery [6]. Despite available therapies, the structural and mechanical properties of injured tendons rarely return to those of healthy tissue [7]. However, recent advances in biomaterials and tissue engineering have introduced new therapeutic avenues for addressing tendon ruptures and tendinopathies [8]. In recent years, tissue engineering strategies have increasingly succeeded in replicating the physiological environment of injured tendons by integrating cells, scaffolds, bioactive molecules, and synthetic constructs. This interdisciplinary approach—merging molecular biology with materials science—holds substantial promise for enhancing tendon regeneration [9]. This review highlights key principles and recent developments in the use of biomaterials and artificial tendon augmentations for tendon regeneration and replacement. It specifically focuses on the application of implanted scaffolds and discusses emerging strategies such as drug delivery through biomaterials and stem cell incorporation at the injury site. The evidence discussed in this review spans in vitro studies, small- and large-animal models, and human investigations, which represent distinct levels of translational maturity. Findings from preclinical models should therefore not be interpreted as direct evidence of clinical efficacy, particularly because differences in tissue scale, physiological loading, and rehabilitation may influence scaffold performance in large animals and humans.

The literature included in this narrative review was identified primarily through searches of PubMed and Google Scholar. Search terms included “tendon injuries,” “biomaterials,” “scaffolds,” “stem cells,” and “artificial tendons,” used individually and in combination. Studies were prioritized based on their relevance to tendon injury and repair, biomaterials and scaffold development, regenerative strategies, and translational or clinical applications.

## 2. Tendon Injuries

### 2.1. Anatomy and Physiology of Tendons

Tendons are key components of the musculoskeletal system, functioning as specialized connective tissues that connect muscles to bones and enable the transmission of mechanical forces required for joint movement [6,10]. Structurally, tendons are highly organized and relatively hypocellular. They connect to muscle via the myotendinous junction, a specialized, highly folded interface characterized by muscle-fiber membrane invaginations, extracellular-matrix components, and associated cellular structures that increase the contact area and reduce local stress concentrations during force transmission [11]. This specialized architecture is relevant to scaffold design, as reconstruction of the myotendinous junction involves distinct biological and mechanical requirements compared with reconstruction of the tendon mid-substance [12].

At the opposite end, tendons attach to bone through a specialized structure known as the enthesis [13]. Entheses are generally classified as fibrous or fibrocartilaginous, which differ in their composition, organization, and mechanical environment. Fibrocartilaginous entheses exhibit a graded transition from tendon to unmineralized fibrocartilage, mineralized fibrocartilage, and bone, providing a functional interface between tissues with markedly different mechanical properties [14]. Consequently, tendon-to-bone healing presents distinct biological and mechanical requirements and should be distinguished from regeneration of a mid-substance tendon defect. The biomechanical function of tendons depends on the precisely regulated interaction between resident cell populations and the composition of the extracellular matrix (ECM). This interplay maintains the tendon’s structural integrity and enables efficient force transmission during routine physical activities [15].

**Figure 1:**
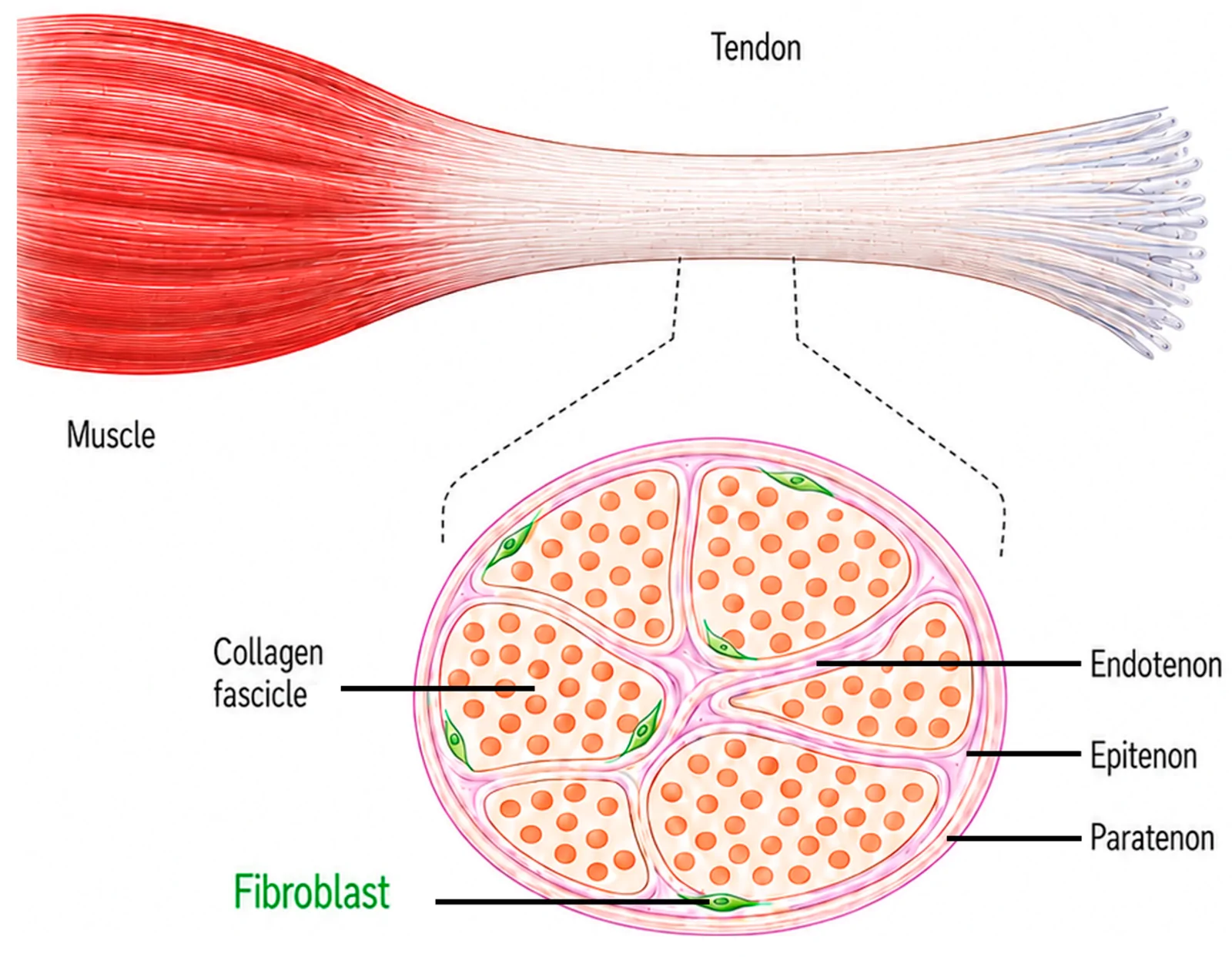
Tendon organization. The tendon tissue is composed of collagen fascicles, which in turn are composed of collagen fibers, made from collagen fibrils. The endotenon, a connective tissue layer, separates individual fascicles and contains blood vessels and nerves. Surrounding the tendon is the epitenon, and in some tendons, an additional paratenon layer of loose connective tissue facilitates gliding between the tendon and surrounding tissues. In other tendons or anatomical regions, a distinct synovial tendon sheath may be present.

Tendon tissue is primarily composed of parallel, densely packed collagen fibers embedded within a well-organized extracellular matrix (ECM), along with resident cells. The main structural component is type I collagen, which consists of a triple helix made up of two α1 chains and one α2 chain. These collagen molecules successively assemble into fibrils, which then form collagen fibers, fascicles (also known as collagen bundles), and ultimately the functional tendon unit [16]. The fascicles are separated by the endotenon, a loose connective tissue that contains fibroblasts, blood vessels, and nerves [10]. The entire tendon is surrounded by the epitenon. Depending on the tendon and anatomical region, the epitenon may be surrounded by a paratenon, or the tendon may pass through a synovial tendon sheath; these structures should not be considered consecutive universal layers. The paratenon is a loose connective tissue layer that facilitates gliding between the tendon and surrounding tissues, whereas a true synovial tendon sheath is a distinct double-layered structure surrounding particular tendons [11,16] (Figure 1).

The predominant cell types within the tendon niche are tenoblasts and tenocytes. Tenocytes are fibroblast-like cells located between collagen fibers and within the endotenon. They are responsible for synthesizing type I collagen and other ECM components, particularly during growth and tissue repair. Rather than representing a simple linear differentiation pathway from tenoblasts to terminally differentiated tenocytes, tendon cells comprise heterogeneous resident stromal populations whose phenotypes and functions may vary according to anatomical location, developmental origin, age, mechanical environment, and injury state. Both resident and extrinsic cell populations may contribute to tendon repair, fibrosis, and matrix remodeling [17,18]. In addition to these mature cell types, tendons also contain tendon stem/progenitor cell (TSPC) niches. These cells exhibit clonogenic potential and self-renewal capacity, contributing to tissue homeostasis and regeneration [19,20,21]. Type I collagen is the predominant ECM protein in tendons, with type III collagen present in smaller quantities. The latter becomes especially important in pathological conditions and during tendon healing. Alongside collagen, the ECM also contains elastin, glycoproteins, and proteoglycans, as well as other specialized molecules such as Cartilage oligomeric matrix protein (COMP), lubricin, and tenascin-C [20].

### 2.2. Epidemiology and Etiology of Tendon Injury

Since tendons transmit forces between muscle and bone during repetitive motion, they are inherently susceptible to both acute and chronic injury [2]. Various injury mechanisms can lead to tendinopathy or tendon rupture, often involving a combination of acute trauma and chronic degeneration. For instance, tendon ruptures may occur due to sudden overload or laceration but are frequently preceded by underlying intrinsic pathology [2]. While injuries such as rupture or laceration result from stresses exceeding physiological limits, repetitive microtrauma—even within normal loading thresholds—can also induce tendinopathy and increase the risk of both acute and chronic tendon damage [21].

Tendon pathologies involve a spectrum of injuries, ranging from acute trauma to chronic degenerative conditions, which differ in their underlying pathology, clinical presentation, and management. Chronic tendon injury, or tendinopathy, is typically associated with persistent pain and functional impairment. While its exact etiology and pathogenesis remain incompletely understood, abnormal mechanical loading is widely recognized as a primary contributing factor [22,23]. In contrast, acute tendon injuries usually involve partial or complete tears resulting from sudden traumatic events [17]. While extrinsic factors—such as sports and physical activity—are commonly associated with tendon injuries, several intrinsic factors also influence an individual’s susceptibility. These include age, sex, underlying systemic diseases (e.g., diabetes mellitus, rheumatoid arthritis), and genetic predisposition [21].

The rotator cuff, Achilles, tibialis posterior, and patellar tendons are among the most commonly affected by tendon pathologies [24]. Epidemiological data show that chronic tendinopathies are far more prevalent than acute tendon ruptures across all major tendons. For example, Achilles tendon ruptures occur at an incidence of approximately 7–11 per 100,000 person-years, typically affecting middle-aged recreational athletes, whereas chronic Achilles tendinopathy affects ~6% of the general population and up to 5% of recreational runners each season [25,26]. Rotator cuff pathology is similarly common, with incident cases estimated at ~87 per 100,000 person-years and age-related degenerative tears affecting more than 20% of adults in their 60s [27].

### 2.3. Biology of Tendon Injury and Repair

Tendons are critical load-bearing structures, and their integrity is tightly regulated by the cellular response to mechanical stimuli. Tenocytes sense mechanical load transmitted through the extracellular matrix (ECM) and, in turn, modulate ECM composition. Mechanical loading is therefore essential for maintaining tendon homeostasis, but excessive or abnormal loading can lead to maladaptive remodeling and degeneration [1]. Several hypotheses have been proposed to explain the etiology of tendinopathy, including hypoxia, ischemic injury, oxidative stress, apoptosis, and inflammatory cytokine production [28,29,30,31,32,33]. These mechanisms are not mutually exclusive and likely interact in a complex manner. Four key models have emerged in the literature:

1. Repetitive physiological loading, such as that occurring during regular exercise, stimulates tendon remodeling and adaptation. However, repeated loading also induces transient matrix degradation, which, when not adequately balanced by subsequent repair, may contribute to accumulation of tendon microdamage. This remodeling process involves both collagen synthesis and degradation, with an initial net degradation phase followed by a shift toward net synthesis [23]. The ECM breakdown seen in early remodeling suggests an important role for matrix metalloproteinases (MMPs) in regulating tendon adaptation [34]. In animal models, physiological exercise enhances cell proliferation, particularly of tendon-derived progenitor cells, and increases collagen production [35]. For example, treadmill running has been shown to increase the presence of myofibroblasts, highlighting their role in remodeling [36].
2. Oxidative stress following ischemia–reperfusion: During high tensile loading, transient ischemia may occur, followed by reperfusion and the generation of reactive oxygen species (ROS). This oxidative stress may contribute to tendinopathy. Supporting this, peroxiredoxin 5, an antioxidant enzyme, has been found to be upregulated in human tenocytes affected by tendinopathy [28,29].
3. Apoptotic pathways induced by strain: Mechanical strain has been shown to trigger signaling cascades involving protein kinases that regulate tenocyte apoptosis, potentially contributing to cellular loss and tissue degeneration [23].
4. Inflammatory cytokine-mediated injury: Inflammatory mediators such as prostaglandin E2 (PGE2) and interleukin-6 (IL-6) have been implicated in the development of tendinopathy in animal models [34,35]. Furthermore, physiological levels of cyclic strain can induce IL-6 secretion by tenocytes, suggesting that even non-pathological loading may contribute to inflammation under certain conditions.

Although the relative contribution of these mechanisms remains under investigation, it is likely that tendinopathy arises from a multifactorial interplay among mechanical, cellular, and molecular stressors [37].

The healing response begins immediately after acute injury with the formation of a hematoma, initiating primary hemostasis. Vascular disruption and the release of growth factors and pro-inflammatory cytokines by intrinsic tendon cells recruit inflammatory cells to the injury site. These immune cells facilitate the breakdown of the clot and clearance of necrotic tissue. Neutrophils and macrophages play a central role during this stage, phagocytosing debris and releasing a second wave of cytokines that drive the transition to the proliferative phase [37,38,39]. During proliferation, granulation tissue is deposited, rich in type III collagen and marked by a peak in DNA content [40]. The final phase—remodeling—is characterized by reduced ECM synthesis, organization of collagen fibers along the tendon’s longitudinal axis, and a gradual normalization of the collagen type III to type I ratio. Matrix metalloproteinases (MMPs) have also been identified as key regulators of ECM remodeling during this phase [38,41]. However, the healing phases overlap and may differ according to the vascularity and anatomical location. Tendon healing involves both intrinsic and extrinsic mechanisms and displays a balance between matrix remodeling and fibrotic scar formation; particularly in flexor tendons, excessive scar formation may cause adhesions and impair tendon gliding [42].

## 3. Biomaterials for Tendon Repair

Tendon injuries, such as tears or ruptures, remain challenging to manage and often result in long-term pain and functional impairment. Because of the inherently low regenerative capacity of tendon tissue, healing rarely restores the original structural and mechanical properties [43,44]. Current clinical repair strategies often rely on autografts, which have limited availability and can cause donor site morbidity. Experimental approaches involve biocompatible polymers, either through surgical reconstruction with artificial tendons, or via tissue-engineered biodegradable scaffolds that induce tendon regeneration. Artificial tendons that have been tested clinically are typically composed of non-absorbable synthetic polymers, which remain permanently at the implantation site to replace tendon tissue. However, these grafts fail to replicate the complex architecture and biomechanical functions of native tendons, and are associated with complications such as foreign body reactions, inflammation, and synovitis [45,46].

**Figure 2:**
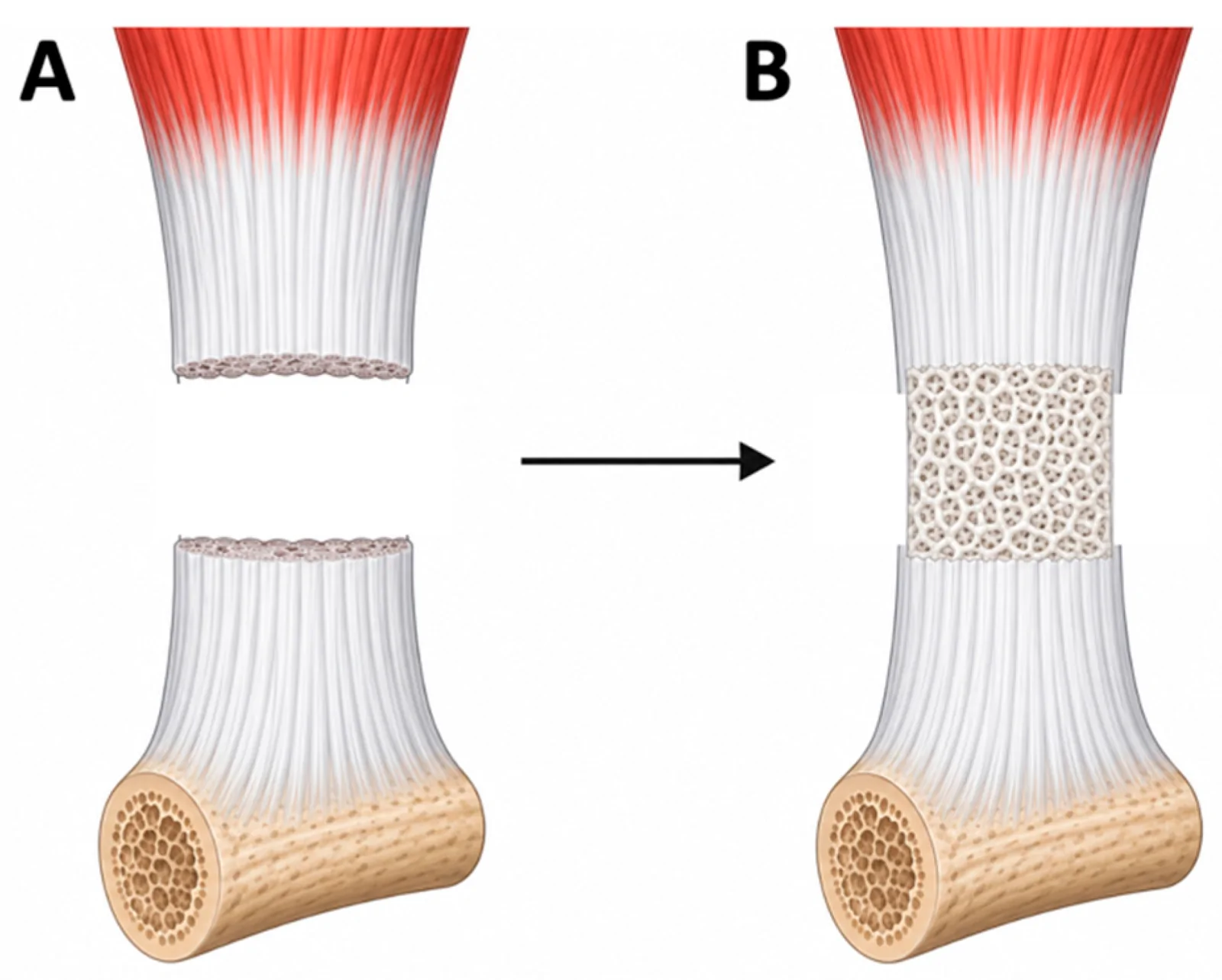
Scaffold implantation at injury site. (**A**) Complex tendon injuries often leave a large tissue gap that cannot be addressed surgically by end-to-end repair. (**B**) Scaffold positioning (simplified) in the gap to facilitate tendon tissue regeneration and organization.

To overcome these limitations, increasing efforts have been directed to biomimetic approaches using tissue engineering and regenerative medicine to construct biodegradable scaffolds for tendon regeneration (Figure 2) [47]. Both biodegradable synthetic and natural polymers have been investigated for scaffold fabrication, with the aim of supporting cell integration and structural mimicry of native tendon tissue to induce tendon regeneration onto the scaffold. Natural biopolymers are attractive due to their excellent biocompatibility and extracellular matrix (ECM)-like properties, whereas synthetic biodegradable polymers provide greater control over mechanical behavior and degradation profiles [48]. More recently, hybrid scaffolds combining natural and synthetic polymers have been developed to balance biocompatibility, degradation kinetics, and mechanical performance. Despite these advances, the selection of optimal biomaterials remains a major challenge, as ideal scaffolds must support cellular activity, deliver growth factors, and provide appropriate mechanical properties and architecture [49].

### 3.1. Natural Biopolymer Scaffolds

Absorbable natural polymers such as collagen, gelatin, silk, and hyaluronic acid are widely used in tendon tissue engineering because they support cell adhesion, proliferation, and differentiation while mimicking the native ECM [48]. Collagen type I scaffolds, especially when combined with mechanical stimulation, have demonstrated enhanced tenogenesis, cell alignment, and collagen deposition [50,51,52]. Gelatin offers additional advantages, including high biocompatibility, low antigenicity, intrinsic cell adhesiveness, and chemical modifiability, making it a versatile scaffold material [53]. Silk, which exhibits relatively high tensile strength among natural polymers, has been applied in fabricating scaffolds for tendon and ligament repair [54]. Hyaluronic acid, with its viscoelasticity, biocompatibility, and anti-inflammatory properties, also promotes tendon regeneration, although its rapid degradation often necessitates chemical modification to improve stability and processing [55].

### 3.2. Synthetic Biopolymer Scaffolds

The most widely used synthetic biopolymers in tendon and interface tissue engineering are aliphatic polyesters, including polylactide (PLA), poly(glycolic acid) (PGA), poly(lactide-co-glycolide) (PLGA), polycaprolactone (PCL), and poly(p-dioxanone) (PPDO). These materials are advantageous due to their scalability, processability, reproducibility, mechanical strength, controlled degradation, and established biocompatibility, with some already FDA-approved for clinical use [48]. Increasingly, hybrid polymer composites are being explored to integrate the biological functionality of natural polymers with the mechanical resilience of synthetic ones. Electrospun hybrid scaffolds, particularly blends of collagen with PCL, PLA, or PLGA, are among the most widely investigated due to their cost-effectiveness, ease of fabrication, and ability to mimic the fibrous architecture of native tendon tissue [56,57].

## 4. Surface Modification

The topographical and mechanical properties of tissue engineering scaffolds critically influence cellular responses relevant to tendon healing, including macrophage immune phenotype, tenocyte proliferation and extracellular matrix (ECM) synthesis, and stem cell tenogenic differentiation [58]. Key scaffold features include fiber diameter, pore size, alignment, surface roughness, and matrix stiffness.

Fiber diameter plays a pivotal role in directing cell behavior. Cardwell et al. investigated electrospun fibers with diameters below 1 µm, between 1 and 2 µm, and above 2 µm. Although scleraxis (Scx) expression was suppressed on all electrospun scaffolds relative to smooth films, it was consistently higher on large-diameter fibers than on medium-diameter fibers [59]. Conversely, smaller fiber diameters promote ECM synthesis and deposition by tenocytes [60]. In a study of human tendon fibroblasts, fiber diameters of 320 nm, 680 nm, and 1.80 µm were evaluated over four weeks. Nanofiber scaffolds supported greater cell numbers and total collagen and proteoglycan production, whereas microfibers promoted the expression of tendon fibroblast phenotypic markers [61]. These findings have led to the hypothesis that nanofibers, which approximate the dimensions of collagen fibrils, may resemble damaged matrix and stimulate cells to deposit new ECM, whereas microfibers, similar in size to collagen fibers, mimic healthy matrix and favor a fibroblast-like phenotype [61]. However, cellular responses to electrospun scaffolds are also influenced by other properties, including polymer composition, fiber density, pore interconnectivity, surface properties, stiffness, and degradation behavior [62]. In vitro studies further indicate that larger fiber diameters increase angiogenic cytokine release by murine macrophages; however, results on macrophage polarization remain inconsistent, highlighting the need for further investigation in tendon injury models [63,64].

Pore size is another determinant of scaffold performance. Caliari and Harley evaluated anisotropic collagen–glycosaminoglycan scaffolds with mean pore sizes of 55 ± 18, 152 ± 25, and 243 ± 29 µm. Tendon cells showed little penetration into the smallest-pore scaffolds but were more evenly distributed throughout scaffolds with pores of 152 and 243 µm [63]. Larger pores generally enhance infiltration and proliferation of tenocytes and stem cells [63,64]. For example, Whitlock et al. demonstrated that increasing the porosity of a tendon-derived scaffold permitted host-cell infiltration while retaining approximately 76–78% of the tensile properties of the original tendon [65]. These findings illustrate that improvements in tissue accessibility must be balanced against preservation of mechanical integrity [64].

Fiber alignment is especially critical, as the anisotropic collagen organization in native tendon provides biomechanical cues essential for tissue-specific function [63,65,66,67,68]. Aligned fibers improve longitudinal cell orientation, proliferation, and metabolic activity in tenocytes [63,67], and upregulate Col I α1 and Tnmd expression in stem cells [65,68], compared with randomly oriented fibers. In anisotropic collagen–glycosaminoglycan scaffolds, tendon cells were significantly more likely to align within ±10° of the scaffold’s longitudinal axis than cells in isotropic controls (*p* < 0.02) [63]. By contrast, disorganized fibers enhance macrophage expression of pro-inflammatory M1 markers such as CCR7 and cytokines such as IL-1β [66]. Similarly, human tendon fibroblasts exposed to pro-inflammatory cytokines reduced tenogenic marker expression and increased matrix-degrading enzyme expression, with this effect exacerbated on disorganized compared to aligned scaffolds [69]. Collectively, these findings suggest that scaffold architectures mimicking native tendon alignment are more conducive to regenerative signaling and pro-healing immune responses. However, increased expression of tenogenic markers alone should not be interpreted as evidence of mechanically functional tendon regeneration.

The role of scaffold surface roughness in tendon repair remains less studied. Although macrophage responses to smooth films and fibrous scaffolds have been investigated, the available studies do not establish an independent, quantitatively defined optimal surface-roughness range for tendon regeneration. Therefore, the effects of roughness should be distinguished from those of fiber diameter, alignment, and other simultaneously varying surface characteristics [70]. Matrix stiffness represents another critical property given the mechanical demands of tendon tissue. Tenocytes on stiffer scaffolds exhibit greater proliferation, metabolic activity, and reduced ECM contraction [71]. However, excessive stiffness can induce macrophage pro-inflammatory cytokine release and impair tendon-derived stem cell differentiation, whereas reduced stiffness favors an anti-inflammatory macrophage phenotype [72,73,74] and promotes tenogenic differentiation of stem cells [75]. Sridharan et al. evaluated macrophages on substrates with stiffness values of 11, 88, and 323 kPa, observing anti-inflammatory-associated responses on the softer substrates and pro-inflammatory-associated responses on the stiffest substrate [72]. Similarly, Blakney et al. investigated hydrogels with compressive moduli of 130, 240, and 840 kPa. Following inflammatory stimulation, macrophages cultured on the stiffest hydrogels exhibited higher IL-1β and IL-6 expression than those cultured on softer substrates [74]. In a separate study, Liu et al. investigated tendon-derived stem cells on hydrogels spanning approximately 2.34 ± 1.48 to 24.09 ± 14.03 kPa, demonstrating stiffness-dependent regulation of differentiation [76]. These stiffness ranges should not be directly compared as universal thresholds because the studies employed different materials, mechanical measurements, and cell populations.

These findings underscore the importance of defining an optimal stiffness range that balances the diverse responses of tenocytes, stem cells, and macrophages to support effective tendon repair. More broadly, scaffold parameters should be evaluated collectively, as improvements in cellular infiltration, longitudinal mechanical performance, or initial stability may involve trade-offs in load-bearing capacity, transverse integrity, and cellular responses.

## 5. Biodegradation

Biodegradability is a critical property in scaffold engineering, as the degradation process must be synchronized with tissue regeneration to maintain sufficient mechanical strength throughout healing. Ideally, the scaffold should provide structural support until the newly formed tissue can withstand physiological loads. Importantly, polymer degradation and loss of mechanical competence do not necessarily occur at the same rate. Molecular chain scission and deterioration of mechanical properties may precede substantial scaffold mass loss. Therefore, degradation should be considered in relation to both material loss and preservation of mechanical function. In addition, the byproducts generated during polymer degradation must be non-toxic and readily eliminated through normal physiological processes to avoid adverse immune reactions [77]. The degradation rate of biopolymer scaffolds is closely related to tissue viability and regenerative outcomes. If scaffold degradation occurs more rapidly than wound healing and native tissue formation, cells may lose the extracellular matrix (ECM)-like support required for proper growth and organization. As a result, tissue regeneration may be incomplete or defective, while accumulated degradation byproducts may not be efficiently cleared from the body [78]. Conversely, excessively slow scaffold degradation can lead to prolonged material retention, scaffold encapsulation, chronic inflammation, and poor integration with the host tissue, potentially resulting in implant rejection [79].

Several factors influence scaffold degradation, including the local biological environment (pH and enzymatic activity), scaffold architecture (porous structures, hydrogels, or bulk materials), polymer composition and functional groups, surface and bulk chemical modifications, implantation site, mechanical loading conditions, and patient-related factors such as age and comorbidities [77]. Scaffold degradation occurs through several mechanisms that may act simultaneously rather than independently. The principal pathways include enzymatic degradation, in which enzymes catalyze polymer breakdown; hydrolytic degradation, involving chain scission by water molecules; oxidative degradation, mediated by reactive oxygen species; and physical degradation resulting from mechanical loading, wear, or swelling [80]. Synthetic polymers commonly used in tissue engineering, particularly polyhydroxy esters, primarily degrade through hydrolytic chain scission [81]. In contrast, natural polymers such as collagen and chitosan possess molecular structures similar to the native ECM and are therefore more susceptible to enzymatic degradation [82]. To better control degradation kinetics, natural and synthetic polymers are often combined to exploit the advantages of both material classes [83].

The degradation rate is also influenced by the scaffold’s surface area-to-volume ratio, which is closely associated with water uptake and wettability. In general, scaffolds with a larger surface area-to-volume ratio degrade more rapidly because they allow greater fluid penetration [84]. Mechanical stimulation further affects degradation behavior. For example, PLGA scaffolds have been shown to degrade more rapidly in shaking incubators than under static conditions, highlighting the role of dynamic loading in accelerating polymer breakdown [85]. In addition, external factors such as ultrasound, heat, and radiation can indirectly alter scaffold degradation by modifying polymer morphology and material properties [86]. Cooke et al. demonstrated that PCL scaffolds degrade more rapidly when exposed to radiation doses commonly used in cancer therapy [87]. The byproducts generated during scaffold degradation can significantly alter the local microenvironment by changing the surrounding pH. Such changes may affect cellular energy metabolism, extracellular matrix synthesis, and overall cell behavior [88]. Acidic conditions have been reported to promote fibroblast proliferation, whereas a more alkaline environment (pH 7–8) is associated with peak protease activity [89].

## 6. Foreign Body Response

The primary goal of biomaterials in tissue engineering is to replace damaged tissues with functional, site-specific constructs that can integrate with the host and restore normal function. Therefore, the host response to the implanted biomaterial dramatically influences the functional outcome. The immune system plays a central role in protecting the body against foreign materials and pathogens while also organizing tissue repair following injury [90]. Consequently, the immune-mediated response to implanted biomaterials, which are inherently recognized as foreign bodies, is of paramount importance for the success of tissue engineering strategies. Historically, immune responses in regenerative medicine were viewed primarily as obstacles to be minimized, as excessive inflammation often led to poor integration, fibrosis, and impaired tissue regeneration. More recently, however, biomaterial design has shifted toward actively modulating the immune response to promote a favorable interaction with the host environment rather than simply suppressing inflammation [91]. This emerging concept of immunomodulatory biomaterials has contributed to the development of more effective regenerative therapies [92].

The immune system continuously monitors the body for pathogens and tissue damage. Upon recognition of a threat, it initiates a coordinated inflammatory cascade. Danger signals released by injured or dying cells, as well as pathogen-associated molecules, are detected by tissue-resident immune cells, which subsequently secrete inflammatory mediators. These signals activate antigen-presenting cells, including dendritic cells and macrophages. During the early stages of the innate immune response, neutrophils rapidly infiltrate the injury site to eliminate pathogens and remove damaged tissue while releasing cytokines that recruit macrophages. Macrophage responses are commonly described using the pro-inflammatory M1 and anti-inflammatory M2 framework; however, this represents a simplified model, as macrophage phenotypes exist along a dynamic continuum and may change over time in response to the local microenvironment. Accordingly, individual markers should not be considered definitive evidence of a specific inflammatory or regenerative macrophage phenotype [90].

In the context of infection, acute injury, or biomaterial implantation, antigens and danger-associated signals are captured by dendritic cells and presented to immature T and B lymphocytes within the lymph nodes. This process initiates the adaptive immune response, leading to the formation of effector T cells, antibody-producing plasma cells, and memory T and B cells that provide long-term immunological protection upon subsequent exposure to the same antigen [90].

Scaffolds designed for tissue engineering often incorporate bioactive molecules or living cells that, following implantation, can stimulate cellular proliferation, influence the phenotype of infiltrating immune cells, and promote tissue regeneration. In addition to their biological components, the physical characteristics of biomaterials have a profound effect on the host immune response. Parameters such as scaffold size, shape, surface topography, charge, and chemical composition can significantly influence innate immune cell behavior. For example, scaffold pore size has been shown to affect fibrotic capsule formation and macrophage polarization.

Larger pore sizes in electrospun scaffolds have been associated with a shift toward markers associated with an anti-inflammatory M2-like macrophage phenotype [93]. However, increasing porosity may reduce the load-bearing capacity of the construct and alter its mechanical behavior relative to native tendon tissue [93]. Similarly, scaffold stiffness and the degree of crosslinking can regulate macrophage polarization and influence the balance between pro-inflammatory and anti-inflammatory responses. Because crosslinking also affects biodegradation, modifications in scaffold chemistry may indirectly alter immune cell recruitment and the overall inflammatory environment. For example, replacing glutaraldehyde with hexamethylene diisocyanate as the crosslinking agent in collagen scaffolds resulted in a tenfold increase in neutrophil recruitment, even after 28 days of subcutaneous implantation [94].

The chemical composition of biomaterials also plays a crucial role in determining the host response. Crosslinking agents such as carbodiimide and glutaraldehyde, although commonly used to improve the mechanical stability of extracellular matrix-based scaffolds, have been shown to promote an early pro-inflammatory immune phenotype and reduce macrophage-mediated scaffold degradation [95,96]. In addition, scaffold geometry and implant size can influence the magnitude of the foreign body reaction and the degree of fibrosis. Studies have demonstrated that simply increasing implant size is insufficient to prevent foreign body responses, whereas spherical implant geometries are more effective in reducing fibrotic encapsulation [97]. Furthermore, degradation products derived from extracellular matrix-based hydrogels have been reported to induce macrophage responses that differ from those elicited by particulate scaffold materials [98]. Importantly, an expected transient foreign-body response should be distinguished from persistent pathological responses, including chronic inflammation, fibrous encapsulation, particle-induced synovitis, and clinically significant implant failure [99].

## 7. Engineered Topographies for Tendon Regeneration

**Figure 3:**
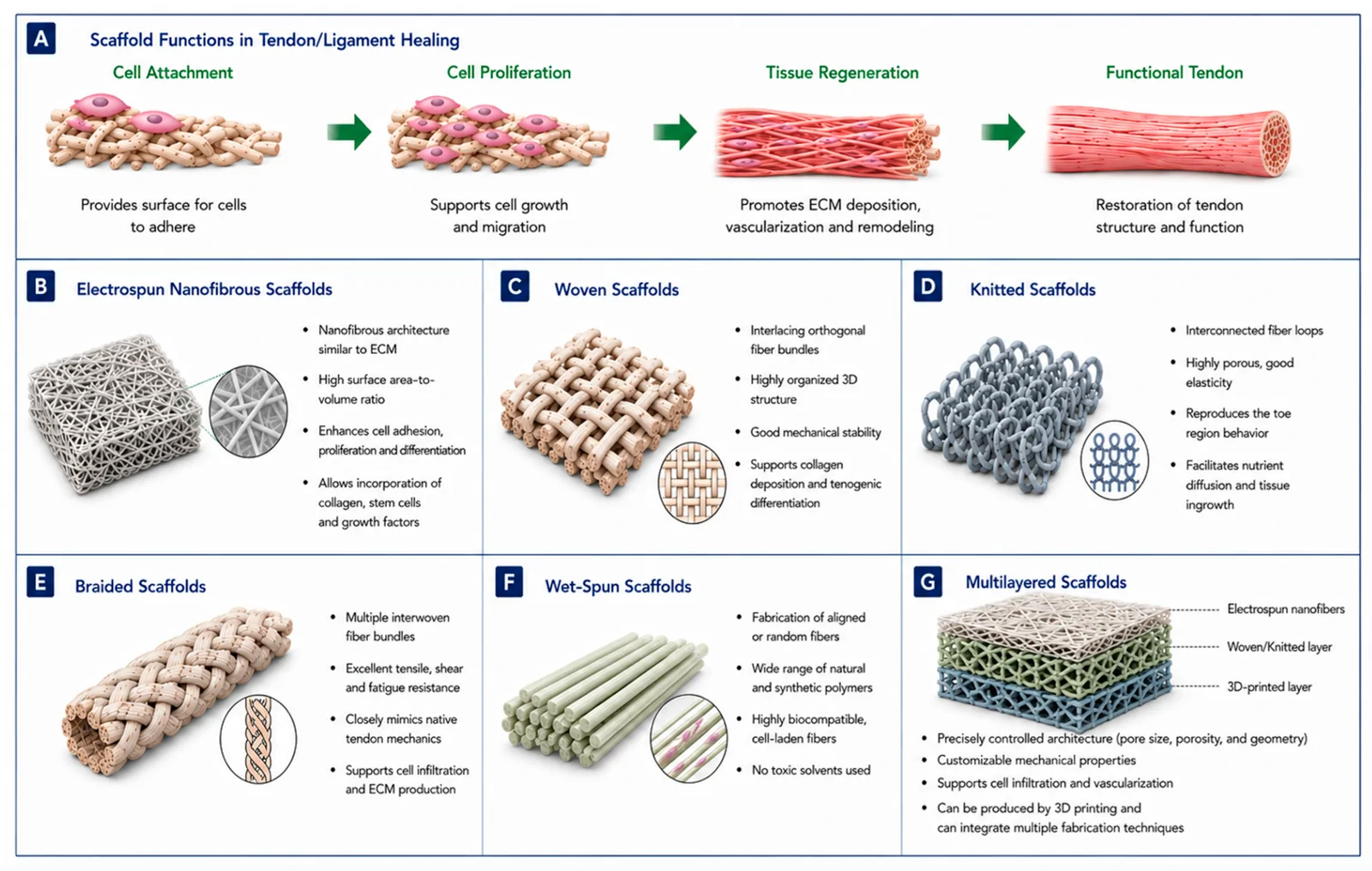
Different types of scaffold topographies. (**A**) Cell attachment onto scaffold and new tendon formation. (**B**) Electrospun scaffolds. (**C**) Woven scaffolds. (**D**) Knitted scaffolds. (**E**) Braided scaffolds. (**F**) Wet-spun scaffolds. (**G**) Multilayered structures.

An ideal engineered tendon construct should provide temporary mechanical support, promote tissue formation, and gradually degrade as the newly formed tissue matures, ultimately allowing complete regeneration of the injured site (Figure 3A) [100]. Beyond these general requirements, scaffold design should consider site-specific geometry, hierarchical alignment, fatigue resistance, suture retention, biocompatibility, appropriate porosity for cell infiltration and matrix deposition, low adhesion formation where tendon gliding is required, and integration with the relevant anatomical interfaces. Importantly, these requirements may conflict. Increased porosity can facilitate tissue infiltration while reducing mechanical strength, greater stiffness can improve initial stability but promote stress shielding, and slower degradation can preserve mechanical support while prolonging foreign-body exposure. Thus, scaffold properties should be balanced according to the anatomical site and intended function rather than optimized independently [101,102]. Notably, a biologically favorable scaffold is not necessarily suitable as a functional load-bearing tendon substitute, as its clinical utility also depends on adequate tensile strength, stiffness, fatigue resistance, viscoelastic behavior, load transfer, and degradation kinetics under physiological loading.

Importantly, the native tendon tissue that regenerates into the scaffold can detect and respond to mechanical, biological, and chemical stimuli [103]. Mechanical stimulation plays a fundamental role in tendon development, repair, and healing [104]. Therefore, reproducing tendon mechanics requires consideration of its anisotropic and viscoelastic behavior, including fiber alignment and response to cyclic loading, rather than matching a single mechanical parameter. Moreover, sufficient mechanical integrity should be maintained as the scaffold degrades and is replaced by newly formed tissue [105]. Therefore, scaffold-related mechanical properties, including surface topography and geometry, can significantly influence cellular behavior and function as also demonstrated in ligament tissue-engineering models [106]. Furthermore, as tendon fibers regenerate, they often follow the directionality of the scaffold, meaning that aligned micro topographies can induce aligned fiber regeneration and assist in reconstructing the native tendon microarchitecture.

The availability of numerous polymers, fabrication techniques, and design strategies has enabled researchers to optimize scaffold dimensions, mechanical performance, biological properties, sterility, and degradation kinetics in an effort to better replicate the complexity of native tendons and ligaments [107]. Evidence from ligament tissue-engineering models, including ACL reconstruction, has also contributed to the development of scaffold design and fabrication strategies; however, these findings should not be considered direct evidence of tendon regeneration because tendons and ligaments differ in their function, loading conditions, anatomical interfaces, and biological behavior [107]. Specifically, fibrous scaffolds are widely used in tendon and ligament tissue engineering because they mimic the anisotropic architecture and collagen alignment of native tissues while providing mechanical support and promoting cellular infiltration during regeneration [105]. However, direct quantitative comparison of mechanical performance between studies is limited by differences in scaffold geometry, dimensions, fabrication methods, testing protocols, and reported mechanical parameters. In the following section, we discuss several important engineered fiber topographies employed in tendon scaffold fabrication (Table 1).

### 7.1. Strand Fibers

Strand fibers were among the first scaffolds investigated for tendon repair. Carbon fibers demonstrated high tensile strength, low elongation, and the ability to support collagen deposition and tissue regeneration [108]. More recently, collagen-based fibers have been developed to better reproduce the fascicular structure of native tendons. However, their poor mechanical properties and rapid degradation have limited their application. In an ACL reconstruction model, composite collagen–silk scaffolds demonstrated favorable mechanical properties but remain limited by incomplete biodegradation [109].

**Figure 4:**
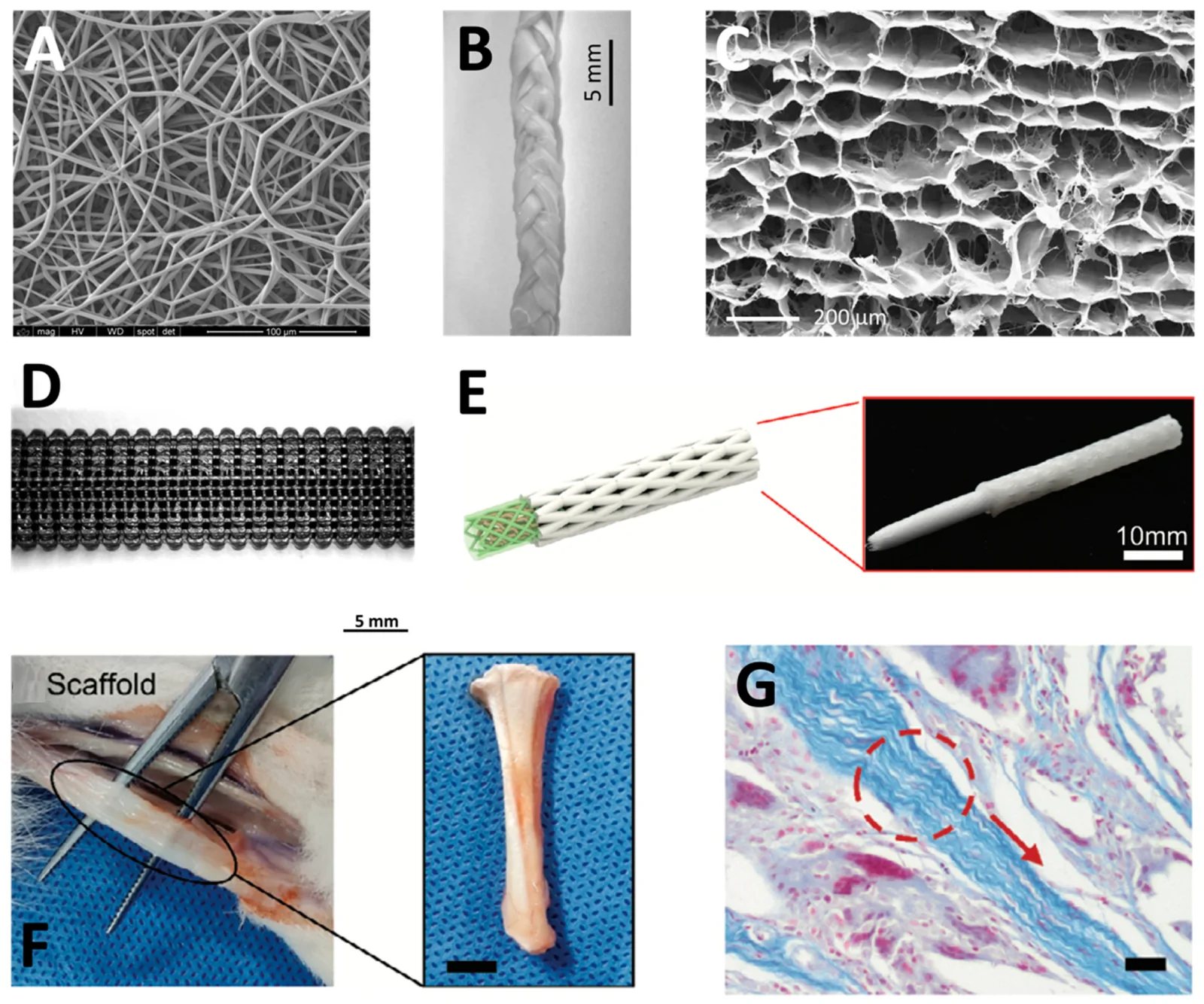
Examples of engineered scaffolds for tendon reconstruction. (**A**) Electrospun nanofiber scaffold composed of polyglycolic acid–poly-L-lactide-co-ε-caprolactone [128]. Reproduced with permission. (**B**) Collagen-BDDGE-elastin gel non-porous braided scaffold. (**C**) Collagen-BDDGE-elastin was fabricated to create a porous shell around the braided scaffold and provide a reservoir of cells to promote regeneration [122]. Reproduced with permission. (**D**) Three-dimensional printed multilayered Polylactic Acid/Graphite tendon scaffold [129]. Reproduced with permission. (**E**) Multiscale biomimetic complex tendon scaffold fabricated by 3D printing. (**F**) Multiscale scaffold integrated into host tendon. (**G**) Collagen deposition within 3D printed scaffold [130]. Reproduced with permission.

Electrospinning (Figure 3B and Figure 4A) is among the most promising techniques to fabricate strand fiber scaffolds for tissue engineering. Their nanofibrous architecture closely resembles the extracellular matrix and provides a high surface area-to-volume ratio that enhances cell adhesion, proliferation, and differentiation [110]. Furthermore, electrospinning enables the incorporation of biological components such as collagen, stem cells, and growth factors to improve scaffold bioactivity and regenerative potential [111,112].

### 7.2. Woven Scaffolds

Woven scaffolds are produced by interlacing orthogonal fiber bundles, creating highly organized three-dimensional structures (Figure 3C). Commercial woven devices fabricated from PET and PTFE have demonstrated good mechanical stability and the ability to restore joint function [113]. Nevertheless, long-term complications such as creep, fatigue, stress shielding, wear debris, and synovitis have limited their clinical use [114]. More recently, collagen-based woven scaffolds have shown promising biological performance, including enhanced collagen deposition and tenogenic differentiation [115].

### 7.3. Knitted Scaffolds

Knitted scaffolds consist of interconnected fiber loops that generate highly porous structures with good elasticity and the ability to reproduce the characteristic toe region of tendon mechanical behavior (D); these properties have also been investigated in ACL reconstruction models [116]. Their porous architecture facilitates nutrient diffusion and tissue ingrowth; however, excessive porosity may compromise mechanical strength and uniform cell distribution. To address these limitations, knitted scaffolds are often combined with hydrogels or electrospun fibers in ligament tissue-engineering models [117]. For example, PLCL knitted scaffolds coated with aligned electrospun microfibers demonstrated uniform cell alignment, proliferation, and extracellular matrix production [118].

### 7.4. Braided Scaffolds

Braided scaffolds are composed of multiple interwoven fiber bundles and exhibit excellent resistance to tensile, shear, and fatigue loading while closely reproducing the mechanical behavior of native tendons and ligaments (Figure 3E and Figure 4B,C) [119]. Although early braided devices made from polypropylene and PTFE improved joint stability, evidence from ligament augmentation devices showed that their use was associated with inflammation, synovitis, and long-term mechanical failure [120]. Consequently, current research focuses on biodegradable braided scaffolds fabricated from PLA, PGA, PLGA, and PCL, which have been investigated for their effects on cellular organization and mechanical properties in ACL tissue-engineering applications [121]. Additionally, porous biocompatible shells have been added around the braided core to provide a reservoir of cells to promote tissue growth [122]. Braid-twisted scaffolds combine fiber twisting and braiding to better replicate the hierarchical organization of tendon tissue. Within individual experimental studies, they exhibit improved tensile strength, greater porosity, and enhanced abrasion resistance [123]. These ACL-based studies also demonstrated promising cellular and biomechanical properties of braid-twisted scaffolds [124].

### 7.5. Wet-Spun Scaffolds

Wet-spun scaffolds (Figure 3F) allow the fabrication of aligned or randomly oriented fibrous constructs from a wide range of natural and synthetic polymers. Their major advantage is the ability to produce highly biocompatible, cell-laden fibers without exposure to toxic solvents [125].

### 7.6. Multilayered Scaffolds

Multilayered scaffolds (Figure 3G and Figure 4D–G) can be produced by multiple fabrication techniques, including electrospinning and 3D printing, to better mimic the structural and functional complexity of native tendons [76,126]. These systems have demonstrated improved cell infiltration, vascularization, mechanical performance, and regenerative outcomes. Representative examples include multilayered constructs combining electrospun fibers with woven or knitted scaffolds, as well as electrospun nanofibers integrated with 3D-printed structures [127].

**Table 1:** Comparison of fiber-based scaffold architectures for tendon tissue engineering.

Scaffold Architecture	Structural Properties	Biological Advantages	Main Limitations/Clinical Considerations	References
Strand fibers	Fiber diameter, orientation, porosity, and mechanical properties can be tailored	ECM-like nanofibrous architecture promotes cell attachment, proliferation, alignment, differentiation, and ECM deposition.	Small pore size may restrict cell infiltration and tissue ingrowth.	[110,111,112]
Woven scaffolds	Provide high structural stability and mechanical strength.	Support collagen deposition and may promote tenogenic differentiation.	Long-term creep, fatigue, stress shielding, permanent deformation, laxity, and wear debris may occur; abrasion-related debris may contribute to synovitis, and cell infiltration can be suboptimal.	[113,114,115]
Knitted scaffolds	Exhibit good elasticity and can reproduce the characteristic toe region of tendon mechanical behavior.	Interconnected pores facilitate nutrient transport and tissue ingrowth; combination with electrospun fibers can enhance cell alignment and ECM production.	High porosity may impair homogeneous cell distribution and may result in inadequate tissue ingrowth.	[116,117,118]
Braided scaffolds	Support high axial loads and provide good tensile, shear, abrasion, and fatigue resistance.	Biodegradable designs support cell attachment, infiltration, and ECM production.	Earlier non-degradable devices were associated with inflammatory reactions, synovitis, pain, degenerative changes, and long-term mechanical deterioration, limiting clinical use.	[119,120,121,122]
Wet-spun scaffolds	Can be fabricated as aligned or randomly oriented fibers and can guide cellular organization.	Enable production of highly biocompatible, cell-laden fibers without exposure to toxic solvents.	Mechanical properties may be insufficient to match native tendon/ligament, limiting their use as standalone load-bearing constructs.	[125]
Multilayered scaffolds	Allow independent tailoring of individual layers and can improve overall mechanical properties.	Can combine cell infiltration, vascularization, ECM-mimetic architecture, and regenerative functions within a single construct.	Evidence remains predominantly preclinical, and architecture-specific clinical limitations have not yet been clearly defined.	[76,126,127]

## 8. Drug Release

Drugs can be delivered to injured tendons through two principal approaches: local or systemic delivery. Local drug delivery involves administering therapeutic agents directly to the injured tissue using biomaterial-based carriers, whereas systemic delivery relies on introducing the drug into the bloodstream to achieve distribution throughout the body [131,132]. Local administration may avoid systemic drug exposure, thereby reducing side effects while allowing higher drug concentrations to be achieved at the injury site. However, local administration is often more technically challenging and may require advanced injectable delivery systems, such as nanoparticles and microparticles, or rely on non-injectable implants, including preformed scaffolds loaded with a desired drug [131].

While the principal advantage of local delivery is the ability to achieve high local drug concentrations while minimizing systemic exposure and off-target effects, a major challenge is achieving an appropriate and controlled degradation rate of the drug-delivery scaffold. An inappropriate degradation rate may result in excessive inflammatory responses, cytotoxicity, and premature or delayed drug release [133]. In addition, the interaction between the drug and its polymeric carrier is a critical design parameter. The binding affinity must be sufficiently strong to prevent premature drug leakage while remaining weak enough to allow controlled drug release at the target site without inducing toxicity [134]. The following section provides an overview of several biomaterial-based carriers used for drug delivery.

### 8.1. Coated Sutures

Postoperative healing following tendon repair is frequently associated with a high risk of re-tear, largely due to the reduced mechanical strength of the tendon tissue surrounding the sutures [135]. Drug-coated sutures serve as localized drug delivery systems without requiring an additional biomaterial carrier. However, their therapeutic capacity is limited because only short lengths of suture are implanted, restricting the total amount of drug that can be delivered. Furthermore, coated sutures generally lack sustained-release properties, resulting in drug availability primarily during the early postoperative phase of tendon healing [136]. Pasternak et al. demonstrated that doxycycline-coated sutures, fabricated using plasma-treated polybutester monofilament sutures, improved the suture-holding capacity of repaired rat Achilles tendons by inhibiting matrix metalloproteinase-mediated tissue degradation and reducing the risk of surgical re-tears [135].

### 8.2. Drug Release from Polymeric Scaffolds

Scaffolds can be implanted or injected into the body and serve as drug delivery platforms. Drugs can be incorporated into scaffolds using several loading strategies, including physical adsorption and chemical modification, thereby concentrating therapeutic agents at the target site and providing a platform for their controlled release [131]. However, locally delivered drugs may still undergo rapid release and clearance due to limitations in drug-loading and retention. Moreover, the binding affinity between the drug and the biomaterial is a critical parameter: it should be sufficiently low to enable effective drug release, yet sufficiently high to prevent uncontrolled release and potential toxicity [137]. In addition to their role as delivery systems, scaffolds should simultaneously provide a suitable substrate for cell attachment, proliferation, differentiation, and migration, while allowing the transport of nutrients, metabolic waste, and biological signaling molecules necessary for cell survival and tissue regeneration. Thus, scaffold-based drug delivery systems must balance drug-loading and release characteristics with the structural and biological requirements necessary to support tendon regeneration.

Advances in polymer chemistry and biotechnology have enabled the fabrication of hierarchical three-dimensional scaffolds capable of simultaneously providing structural support and sustained localized drug delivery while closely mimicking the native tendon architecture [138]. However, their limited biodegradability may restrict their clinical applicability, particularly when degradation products cannot be efficiently metabolized or eliminated [139]. Hu et al. developed an electrospun scaffold incorporating ibuprofen that effectively reduced inflammation and prevented adhesion formation in a chicken flexor tendon model, demonstrating the therapeutic potential of controlled drug release from polymeric scaffolds [140].

Natural polymers provide an alternative approach and are particularly attractive because of their biocompatibility, biodegradability, and ability to reproduce aspects of the native extracellular matrix. However, achieving sustained and predictable drug release from these materials remains challenging. Drug release from natural polymer scaffolds is often governed primarily by passive diffusion and desorption, resulting in an initial burst release that may expose surrounding cells to excessive drug concentrations and increase the risk of cytotoxicity [20]. This initial burst may also rapidly deplete the incorporated therapeutic agent, shortening the duration over which an effective local concentration can be maintained. Drug-release behavior is further influenced by scaffold properties such as porosity, degradation rate, polymer composition, and drug–polymer interactions, making precise control of release kinetics particularly important. Strategies that modify drug–polymer interactions or scaffold architecture may therefore help reduce burst release and extend therapeutic exposure at the repair site. Consequently, further optimization of natural scaffold composition, drug-loading strategies, and release kinetics is required to maximize their therapeutic potential.

Overall, polymeric scaffolds provide several important advantages as drug delivery systems, including prolonged drug bioavailability, sustained therapeutic effects, and reduced systemic side effects. Importantly, they can combine controlled drug delivery with the structural and biological functions required for tendon tissue engineering, allowing a single platform to both support regenerating tissue and modulate the local healing environment. Nevertheless, this increased functionality is accompanied by several practical limitations. Preformed scaffolds generally require surgical implantation, which increases procedural complexity and may cause additional tissue trauma. Non-degradable or slowly degrading constructs may also require subsequent surgical removal, exposing patients to additional risks, including tissue damage, excessive scar formation, infection, and other postoperative complications [141]. Therefore, the clinical utility of scaffold-based drug delivery depends on achieving an appropriate balance between mechanical support, biocompatibility, degradation, drug-loading capacity, and controlled release while minimizing the invasiveness associated with scaffold implantation. However, the biological and drug-delivery benefits demonstrated in experimental models should not be interpreted as evidence of established clinical efficacy, and further validation in human studies is required before these approaches can be routinely applied to tendon repair.

### 8.3. Biomaterial Nanoparticles for Drug Delivery

Nanoparticles represent a versatile approach for local drug delivery to injured tendons and can be administered directly to the injury site or incorporated into scaffolds and other biomaterial-based delivery systems [142]. Their small size and highly tunable physicochemical properties allow their composition, surface characteristics, and drug-loading capacity to be engineered to regulate drug retention and release kinetics. When incorporated into scaffolds, nanoparticles can provide sustained and localized delivery of therapeutic agents while simultaneously contributing to the biological and physicochemical properties of the scaffold [142]. Alternatively, injectable nanoparticle-based systems can deliver therapeutic agents directly to the injured tendon without requiring implantation of a preformed scaffold. These approaches may improve local drug bioavailability and prolong therapeutic exposure while reducing the rapid diffusion and clearance associated with conventional local drug administration. Nevertheless, local nanoparticle delivery remains limited by factors such as tissue damage associated with repeated injections, potential burst release, and the difficulty of maintaining therapeutic drug concentrations throughout the healing period.

In addition to their use for local administration, nanoparticles may enable systemic administration while retaining preferential drug localization at the tendon injury site. Their size, surface characteristics, and composition can be engineered to regulate drug loading, release kinetics, and circulation time, thereby reducing off-target toxicity [143]. Furthermore, nanoparticle-based delivery systems can improve drug biodistribution, enhance drug solubility and stability, prolong systemic circulation, and facilitate transport across biological barriers [144]. Tissue-targeted nanoparticle delivery therefore represents a potentially non-invasive strategy for achieving localized tendon therapy following systemic administration [145]. However, tendon-specific targeting remains challenging because well-defined molecular targets have not yet been established [146]. Potential strategies include the development of stimuli-responsive nanoparticles that exploit local changes in redox potential, enzymatic activity, and pH. Such approaches require a precise understanding of the tendon healing microenvironment and its temporal changes throughout the healing process [2,38]. For example, hypoxia and changes in the local redox environment during the inflammatory phase may provide stimuli for responsive nanoparticle systems, whereas altered or denatured collagen during subsequent phases of healing may provide a molecular target for surface-functionalized nanoparticles [42,147,148,149,150].

Although targeted nanoparticle-based drug delivery has advanced considerably over the past decade, relatively few studies have investigated its application in tendon regeneration. One notable approach utilized collagen hybrid peptide (CHP)-modified PLGA nanoparticles to deliver rapamycin to injured tendons following subcutaneous administration [144]. By targeting denatured collagen within the damaged tendon, these nanoparticles preferentially accumulated at the injury site while minimizing localization in adjacent healthy tissue. This approach provided sustained rapamycin release, inhibited endochondral ossification, and preserved drug bioactivity. Such studies demonstrate the potential of nanoparticles to bridge local and systemic drug-delivery strategies by allowing a therapeutic agent administered outside the injury site to preferentially accumulate within damaged tendon tissue. However, further identification of tendon-specific molecular targets and characterization of the changing healing microenvironment will be required before targeted systemic nanoparticle delivery can be reliably translated to clinical tendon regeneration.

## 9. Stem Cells

Mesenchymal stromal cells (MSCs) have emerged as one of the most promising therapeutic strategies for tendon repair and regeneration. Numerous experimental and clinical studies have demonstrated their ability to enhance tendon healing through multiple complementary mechanisms [151,152,153]. First, MSCs possess multilineage differentiation potential and can differentiate into tenocyte-like cells. More importantly, they exert potent paracrine effects by secreting cytokines, growth factors, and extracellular vesicles that stimulate angiogenesis, cell proliferation, extracellular matrix (ECM) synthesis, and tissue remodeling [154]. In addition, MSCs exhibit immunomodulatory properties that attenuate excessive inflammation and promote a regenerative microenvironment [155]. Their widespread availability from multiple tissue sources, including bone marrow, adipose tissue, placenta, and umbilical cord, further supports their clinical potential [156]. However, despite encouraging experimental findings and early clinical investigation, the evidence supporting MSC-based therapies for tendon regeneration remains predominantly preclinical, and their clinical efficacy has not yet been consistently established.

MSCs can be readily isolated, expanded, and administered either by direct injection or in combination with biomaterial-based scaffolds and hydrogels. Following tendon injury, MSCs migrate toward the damaged tissue, where they secrete bioactive molecules that regulate cell signaling, stimulate resident tenocytes and tendon stem/progenitor cells (TSPCs), and promote tissue repair [157,158,159]. Consequently, numerous studies have investigated the therapeutic application of exogenous MSCs using intravenous injection, local administration, or tissue-engineered constructs [160,161,162]. For example, Smith et al. demonstrated that bone marrow-derived MSC (BMSC) injection into injured racehorse tendons significantly improved tendon biomechanics, morphology, and ECM organization after six months of treatment [163].

In addition to exogenous stem cells, tendons contain a small population of resident tendon stem/progenitor cells (TSPCs) that are responsible for maintaining tissue homeostasis and supporting repair following injury. Similar to other stem cell populations, TSPCs possess self-renewal capacity and multilineage differentiation potential. However, injury-induced alterations in the tendon microenvironment may promote aberrant differentiation toward chondrogenic, osteogenic, or adipogenic lineages, ultimately compromising tendon regeneration [156]. Therefore, current regenerative strategies aim not only to deliver exogenous MSCs but also to stimulate endogenous TSPCs and enhance their differentiation toward the tenogenic lineage [164].

Increasing evidence suggests that many of the therapeutic effects of MSCs are mediated through extracellular vesicles, including small extracellular vesicles (sEVs), rather than direct cellular engraftment [165,166]. MSC-derived EVs contain proteins, RNAs, microRNAs, and other bioactive molecules that regulate cell signaling through receptor-ligand interactions, membrane fusion, or endocytosis [167]. These extracellular vesicles have demonstrated important roles in immune regulation, angiogenesis, apoptosis, and tissue regeneration, making them an attractive cell-free therapeutic alternative [166,168,169]. However, the therapeutic benefits of MSC-derived EVs in tendon regeneration have been demonstrated primarily in experimental models, and evidence of clinical efficacy in humans remains limited.

One of the major advantages of MSC-derived EVs is their ability to modulate the inflammatory response following tendon injury. During the inflammatory phase, immune cells including macrophages infiltrate the injured tendon and initiate tissue remodeling through the secretion of inflammatory cytokines [170]. MSCs respond to inflammatory signals by releasing immunomodulatory mediators, including TGF-β1, IL-6, and prostaglandin E2 (PGE2), which suppress pro-inflammatory immune cells such as Th1 cells, Th17 cells, and M1 macrophages while promoting the anti-inflammatory M2 phenotype [171]. Furthermore, MSC-derived EVs reduce inflammatory cell infiltration and regulate macrophage polarization through the delivery of microRNAs such as miR-23a-3p, resulting in decreased expression of M1-associated markers (iNOS, IL-6, IL-1β, and TNF-α) and increased expression of M2-associated markers (CD163, IL-10, TGF-β, and arginase-1) [172,173,174].

Besides their immunomodulatory effects, MSCs contribute significantly to angiogenesis and extracellular matrix remodeling. Tendon injury creates a hypoxic microenvironment that disrupts ECM homeostasis and limits tissue regeneration [175,176]. MSCs promote neovascularization through the secretion of vascular endothelial growth factor (VEGF), thereby enhancing endothelial cell proliferation and differentiation [177,178,179]. In addition, MSC-derived EVs transfer angiogenic microRNAs, including miR-30b, to endothelial cells, facilitating vascular growth through the regulation of Delta-like ligand 4 (DLL4) signaling [180,181].

During the proliferative and remodeling phases of tendon healing, MSCs further promote regeneration by stimulating tenocyte proliferation, collagen synthesis, and extracellular matrix production [182]. Because tendon healing is limited by low cellularity and poor vascularity, regeneration is often incomplete, and mechanical properties are rarely fully restored [2]. TSPCs contribute to tendon repair by replenishing the tenocyte population and differentiating into tendon-forming cells [183,184]. Accordingly, current therapeutic strategies increasingly focus on combining exogenous MSC therapy with the activation of endogenous TSPCs through the sustained release of growth factors and other bioactive molecules to maximize tendon regeneration [164].

## 10. Commercial Tendon Augmentations in Clinical Use

Tendon injuries are among the most common conditions encountered in orthopedic practice, and their clinical burden continues to increase with the aging population and growing participation in sports activities [185]. Conventional surgical techniques, including tendon suturing and tendon-to-bone fixation, may provide suboptimal outcomes in chronic tendon disorders [186]. In severe injuries, reconstruction using autologous or allogeneic tendon grafts may therefore be required [187]. However, the availability of cadaveric donor tissue is limited, and tissue banking programs may be unable to meet the increasing demand for tendon grafts [186]. Consequently, several commercial tendon augmentation products have been developed to provide biological support and appropriate viscoelastic and mechanical properties during tendon healing (Table 2). These products derive many of their features from advances in preclinical development of engineered tendon scaffolds and can be broadly classified into three categories: natural, synthetic, and hybrid biomaterial-based products [188].

### 10.1. Natural Biomaterial-Based Products

Biologically derived scaffolds offer several advantages, including biocompatibility, structural similarity to the host extracellular matrix (ECM), and the ability to support cellular attachment and growth. Commercial biological scaffolds are commonly derived from bovine or porcine tissues that undergo extensive processing and purification. Examples include the DX Reinforcement Matrix, derived from porcine dermal ECM, and REGENETEN, composed of bovine type I collagen [189].

Despite approximately a decade of clinical availability, evidence supporting the DX Reinforcement Matrix remains limited. A matched-pair comparative pilot study evaluated arthroscopic supraspinatus repair with porcine dermal patch augmentation (n = 20) against repair alone (n = 20) in patients aged 60 years or older. At 24 months, recurrent tendon defects were identified in 9 of 19 augmented shoulders and in 4 of 20 controls (relative risk 2.4; *p* = 0.096), and no statistically significant between-group differences were observed in postoperative pain or in patient-reported outcome scores [190]. Because the study was small and was not designed or powered as an equivalence or non-inferiority trial, these data indicate an absence of demonstrated benefit rather than evidence that augmented and non-augmented repair are equivalent.

The REGENETEN implant is a highly porous graft composed of type I collagen derived from highly purified bovine Achilles tendon. The implant provides a biological environment that promotes the formation of new tendon-like tissue and augmentation of the existing tendon before being gradually resorbed within approximately six months [191]. In a prospective multicenter study of 33 patients with partial-thickness rotator cuff tears, treatment with REGENETEN was reported to be safe and effective across different tear grades and locations at two years of follow-up [192]. VersaWrap differs from other biologically derived products in both its material source and composition. It is a plant-based bioresorbable hydrogel composed of hyaluronic acid and alginate derived through seaweed fermentation. Most published studies investigating VersaWrap have focused on its application in compressive neuropathies, and no clinical studies evaluating its use in tendon repair have yet been published [189,193,194]. Nevertheless, its bioresorbable hydrogel composition and ability to provide a physical interface between adjacent tissues provide a rationale for its potential use in tendon surgery, particularly where limiting postoperative adhesions and preserving tendon gliding are important. Its relevance to tendon repair therefore remains primarily conceptual and requires direct preclinical and clinical evaluation [195].

### 10.2. Synthetic Biomaterial-Based Products

Synthetic tendon augmentation products primarily rely on the mechanical properties of their constituent polymers to provide temporary structural support during tissue healing. Commonly used synthetic materials include polyethylene, polyester, poly-L-lactic acid (PLLA), polyurethane–urea (PUU), and polycaprolactone (PCL), either alone or in combination with other materials [189]. X-Repair is a biocompatible PLLA mesh characterized by high strength and stiffness. It is designed to be sutured over the damaged tissue to reinforce tendons and other soft tissues while allowing tissue integration. The slow degradation of PLLA provides prolonged mechanical support during the natural repair process before complete scaffold resorption. In an uncontrolled case series of 18 patients undergoing X-Repair-augmented rotator cuff repair, the repair was intact on ultrasound in 15 of 18 patients (83%) at 12 months and in 14 of 18 (78%) at a mean of 42 months, and the mean American Shoulder and Elbow Surgeons score rose from 25 before surgery to 71 at 12 months [196]. As no control group was included, these proportions describe the course of the treated cohort and do not quantify the effect attributable to the device.

BioFiber is a bilayer synthetic absorbable woven scaffold composed of poly-4-hydroxybutyrate (P4HB) fibers and is intended to reinforce soft tissues in conjunction with sutures or suture anchors during tendon repair. Although BioFiber entered the market in 2011, clinical evidence remains limited. A post-market observational study reported that BioFiber-augmented arthroscopic repair represented a promising treatment option for patients with full-thickness rotator cuff tears [197]. The Leeds–Kuff Patch and Pitch–Patch are PET-based augmentation devices introduced in 2012 and 2021, respectively. The Pitch–Patch additionally incorporates a reinforced border and prepared reinforced holes to facilitate suture fixation. A non-randomized interventional study evaluating the Leeds–Kuff Patch demonstrated improvements in clinical outcomes, with patch repair providing greater improvements across outcomes measured by the Oxford Shoulder Score [189]. Similarly, Smolen et al. evaluated Pitch–Patch augmentation for massive rotator cuff tears in a prospective cohort study and reported favorable clinical outcomes together with a substantially reduced retear rate [198].

FlexBand consists of degradable fibers composed of PCL and PUU knitted into a porous matrix designed to provide elasticity, mechanical strength, and biological integration with connective tissue. The scaffold is designed to approximate the mechanical strength of ankle ligaments and tendons and gradually degrades over approximately 4–6 years as it is replaced by newly formed connective tissue [199]. Mendes de Carvalho et al. reported successful treatment of a chronic Achilles tendon rupture using flexor hallucis longus (FHL) transfer reinforced with FlexBand, with improved recovery and strength and complete graft integration at 12 months [200]. Similarly, Solan et al. reported favorable clinical outcomes following FlexBand augmentation of an extensor hallucis longus (EHL) tendon repair [199].

### 10.3. Hybrid Biomaterial-Based Products

Hybrid tendon augmentation products aim to combine the biological advantages of natural biomaterials with the mechanical properties of synthetic polymers, thereby providing an ECM-like environment while maintaining adequate structural support [189]. Since 2009, only two commercial hybrid products have been identified. BioFiber CM, introduced in 2015, combines the synthetic BioFiber scaffold with a bovine collagen coating. The available clinical evidence is limited to the same post-market observational study that evaluated the corresponding scaffold without collagen coating [63]. BioBrace is another hybrid scaffold composed of highly porous type I collagen reinforced with bioresorbable PLLA microfilaments. To date, no published clinical studies evaluating this product have been identified [189]. Its inclusion in the context of tendon repair is based on its combination of a porous collagen matrix intended to support tissue integration with PLLA microfilaments that provide mechanical reinforcement. This hybrid design may address the need for simultaneous biological integration and temporary mechanical support; however, its effectiveness in tendon regeneration remains to be established clinically [201].

**Table 2:** Commercial biomaterial-based products for tendon repair and augmentation.

Product	Composition	Degradability	Intended Anatomical Indication	References
DX Reinforcement Matrix	Acellular porcine dermal ECM	Biologic/remodelable matrix	Rotator cuff/soft-tissue reinforcement	[186,187]
REGENETEN Bioinductive Implant	Highly purified bovine type I collagen	Bioresorbable	Primarily rotator cuff; additional tendon applications	[188,189]
VersaWrap	Hyaluronic acid/alginate-based bioresorbable hydrogel	Bioresorbable	Tendon protection/management; also marketed for nerve applications	[186,190,191]
X-Repair	Poly-L-lactic acid (PLLA) mesh	Slowly bioresorbable	Soft-tissue/tendon reinforcement, including rotator cuff	[192]
BioFiber Scaffold	Woven poly-4-hydroxybutyrate (P4HB)	Bioresorbable	Soft-tissue reinforcement, including tendon repair	[193]
Leeds-Kuff Patch	Polyethylene terephthalate (PET)	Non-bioresorbable	Rotator cuff reinforcement	[186]
Pitch-Patch	PET with reinforced border and reinforced suture holes	Non-bioresorbable	Massive/large rotator cuff repair augmentation	[186,194]
FlexBand	Porous knitted PCL/PUU fibers	Bioresorbable	Tendon and ligament reinforcement; reported Achilles and EHL applications	[196,197]
BioFiber CM	P4HB BioFiber scaffold with bovine collagen coating	Bioresorbable synthetic component with biological collagen coating	Soft-tissue/tendon reinforcement	[186]
BioBrace	Highly porous type I collagen reinforced with PLLA microfilaments	Bioresorbable	Tendon/ligament and other soft-tissue augmentation, including rotator cuff	[186]

## 11. Summary

Tendon injuries continue to represent a major challenge because of the inability of current treatments to fully restore the native structure and function of injured tendons. While surgical repair and tendon grafting remain the standard therapeutic option, complications such as donor-site morbidity and limited graft availability emphasize the need for more effective regenerative therapies. In recent years, significant advances in tissue engineering have expanded the possibilities for tendon healing and regeneration.

The rise of scaffolds, natural and synthetic biomaterials, and surface modification techniques has facilitated the development of constructs that mimic the biological and mechanical characteristics of native tendon tissue. In addition, mesenchymal stromal cell-based therapies, controlled drug delivery systems, and immunomodulatory biomaterials have shown significant promise to promote angiogenesis, improve ECM remodeling, and regulate inflammation during tendon healing. However, the evidence supporting many of these approaches remains predominantly preclinical and encouraging biological outcomes have not yet been consistently translated into restoration of native tendon function in humans. Among the approaches discussed, some commercial augmentation scaffolds have been evaluated clinically, although the available studies are generally limited in size and follow-up, whereas cell-based therapies, targeted drug-delivery systems, and many engineered scaffolds remain supported primarily by preclinical or preliminary feasibility evidence.

Despite these advances, several important limitations continue to restrict the clinical translation of tendon regenerative technologies. Key challenges include maintaining long-term mechanical durability while achieving controlled degradation, ensuring appropriate host integration while minimizing adverse inflammatory and foreign-body responses, and establishing reproducible and standardized manufacturing processes, particularly for complex biofabricated, cell-based, and extracellular vesicle-based therapies. In addition, the long-term safety of many emerging approaches remains insufficiently characterized, and robust human clinical evidence remains limited.

Future research should focus on addressing these translational barriers while also improving vascularization and the integration of regenerative constructs with native tendon tissue. Successful tendon regeneration requires not only tissue formation but also restoration of tissue continuity, hierarchical architecture, integration with the relevant anatomical interfaces, mechanical competence, and functional performance. Accordingly, increased collagen deposition, favorable tenogenic marker expression, or short-term imaging improvements should not, in isolation, be interpreted as evidence of functional tendon regeneration. Furthermore, well-designed clinical trials are essential to establish the long-term efficacy and safety of these new regenerative strategies.

Despite recent progress, there are no currently available biomaterials capable of fully reproducing the biological and biomechanical properties of native tendon tissue. The integration of stromal cell biology, advanced biofabrication technologies, biomaterial science, tissue engineering and drug delivery provides a framework for the continued development of tendon regenerative strategies. However, substantial anatomical, biological, and mechanical barriers remain before many of these approaches can achieve reliable clinical translation and functional regeneration. For many emerging technologies, progression toward routine clinical use will therefore require further validation in large-animal models and well-designed human studies, together with demonstration of reproducible manufacturing, safety, and long-term performance. Importantly, future studies should determine whether promising biological and histological findings translate into durable biomechanical restoration under physiologically relevant loading and, ultimately, clinically meaningful functional improvement.

## Data Availability

No new data were created or analyzed in this study. Data sharing is not applicable to this article.

## References

[1] Andarawis-Puri N., Flatow E.L., Soslowsky L.J. (2015). Tendon basic science: Development, repair, regeneration, and healing. J. Orthop. Res..

[2] Thomopoulos S., Parks W.C., Rifkin D.B., Derwin K.A. (2015). Mechanisms of tendon injury and repair. J. Orthop. Res..

[3] Zafar M.S., Mahmood A., Maffulli N. (2009). Basic science and clinical aspects of Achilles tendinopathy. Sports Med. Arthrosc. Rev..

[4] Rees J.D., Wilson A.M., Wolman R.L. (2006). Current concepts in the management of tendon disorders. Rheumatology.

[5] Haasters F., Polzer H., Prall W.C., Saller M.M., Kohler J., Grote S., Mutschler W., Docheva D., Schieker M. (2011). Bupivacaine, ropivacaine, and morphine: Comparison of toxicity on human hamstring-derived stem/progenitor cells. Knee Surg. Sports Traumatol. Arthrosc..

[6] Gaut L., Duprez D. (2016). Tendon development and diseases. WIREs Dev. Biol..

[7] Lipman K., Wang C., Ting K., Soo C., Zheng Z. (2018). Tendinopathy: Injury, repair, and current exploration. Drug Des. Devel. Ther..

[8] Wu F., Nerlich M., Docheva D. (2017). Tendon injuries: Basic science and new repair proposals. EFORT Open Rev..

[9] Kishore V., Bullock W., Sun X., Van Dyke W.S., Akkus O. (2012). Tenogenic differentiation of human MSCs induced by the topography of electrochemically aligned collagen threads. Biomaterials.

[10] Benjamin M., Ralphs J.R. (2000). The cell and developmental biology of tendons and ligaments. Int. Rev. Cytol..

[11] Tidball J.G., Lin C. (1989). Structural changes at the myogenic cell surface during the formation of myotendinous junctions. Cell Tissue Res..

[12] Snow F., O’Connell C., Yang P., Kita M., Pirogova E., Williams R.J., Kapsa R.M.I., Quigley A. (2024). Engineering interfacial tissues: The myotendinous junction. APL Bioeng..

[13] Benjamin M., Kumai T., Milz S., Boszczyk B.M., Boszczyk A.A., Ralphs J.R. (2002). The skeletal attachment of tendons—Tendon ‘entheses’. Comp. Biochem. Physiol. A Mol. Integr. Physiol..

[14] Apostolakos J., Durant T.J.S., Dwyer C.R., Russell R.P., Weinreb J.H., Alaee F., Beitzel K., McCarthy M.B., Cote M.P., Mazzocca A.D. (2014). The enthesis: A review of the tendon-to-bone insertion. Muscles Ligaments Tendons J..

[15] Lin T.W., Cardenas L., Soslowsky L.J. (2004). Biomechanics of tendon injury and repair. J. Biomech..

[16] Screen H.R.C., Berk D.E., Kadler K.E., Ramirez F., Young M.F. (2015). Tendon functional extracellular matrix. J. Orthop. Res..

[17] Docheva D., Müller S.A., Majewski M., Evans C.H. (2015). Biologics for tendon repair. Adv. Drug Deliv. Rev..

[18] Chuen F.S., Chuk C.Y., Ping W.Y., Nar W.W., Kim H.L., Ming C.K. (2004). Immunohistochemical characterization of cells in adult human patellar tendons. J. Histochem. Cytochem..

[19] Bi Y., Ehirchiou D., Kilts T.M., Inkson C.A., Embree M.C., Sonoyama W., Li L., Leet A.I., Seo B.-M., Zhang L. (2007). Identification of tendon stem/progenitor cells and the role of the extracellular matrix in their niche. Nat. Med..

[20] Riley G. (2004). The pathogenesis of tendinopathy. A molecular perspective. Rheumatology.

[21] Nakama L.H., King K.B., Abrahamsson S., Rempel D.M. (2007). Effect of repetition rate on the formation of microtears in tendon in an in vivo cyclical loading model. J. Orthop. Res..

[22] Kaux J.-F., Forthomme B., Le Goff C., Crielaard J.-M., Croisier J.-L. (2011). Current opinions on tendinopathy. J. Sports Sci. Med..

[23] Magnusson S.P., Langberg H., Kjaer M. (2010). The pathogenesis of tendinopathy: Balancing the response to loading. Nat. Rev. Rheumatol..

[24] Nourissat G., Berenbaum F., Duprez D. (2015). Tendon injury: From biology to tendon repair. Nat. Rev. Rheumatol..

[25] Čretnik A., Košir R. (2023). Incidence of Achilles tendon rupture: 25-year regional analysis with a focus on bilateral ruptures. J. Int. Med. Res..

[26] Lagas I.F., Fokkema T., Verhaar J.A.N., Bierma-Zeinstra S.M.A., van Middelkoop M., de Vos R.-J. (2020). Incidence of Achilles tendinopathy and associated risk factors in recreational runners: A large prospective cohort study. J. Sci. Med. Sport.

[27] White J.J.E., Titchener A.G., Fakis A., Tambe A.A., Hubbard R.B., Clark D.I. (2014). An epidemiological study of rotator cuff pathology using The Health Improvement Network database. Bone Jt. J..

[28] Goodship A.E., Birch H.L., Wilson A.M. (1994). The pathobiology and repair of tendon and ligament injury. Vet. Clin. N. Am. Equine Pract..

[29] Wang M.X., Wei A., Yuan J., Clippe A., Bernard A., Knoops B., Murrell G.A. (2001). Antioxidant enzyme peroxiredoxin 5 is upregulated in degenerative human tendon. Biochem. Biophys. Res. Commun..

[30] Birch H.L., Rutter G.A., Goodship A.E. (1997). Oxidative energy metabolism in equine tendon cells. Res. Vet. Sci..

[31] Arnoczky S.P., Tian T., Lavagnino M., Gardner K., Schuler P., Morse P. (2002). Activation of stress-activated protein kinases (SAPK) in tendon cells following cyclic strain: The effects of strain frequency, strain magnitude, and cytosolic calcium. J. Orthop. Res..

[32] Skutek M., van Griensven M., Zeichen J., Brauer N., Bosch U. (2001). Cyclic mechanical stretching enhances secretion of interleukin 6 in human tendon fibroblasts. Knee Surg. Sports Traumatol. Arthrosc..

[33] Stone D., Green C., Rao U., Aizawa H., Yamaji T., Niyibizi C., Carlin G., Woo S.L.Y. (1999). Cytokine-induced tendinitis: A preliminary study in rabbits. J. Orthop. Res..

[34] Sun H.B., Andarawis-Puri N., Li Y., Fung D.T., Lee J.Y., Wang V.M., Basta-Pljakic J., Leong D.J., Sereysky J.B., Ros S.J. (2010). Cycle-dependent matrix remodeling gene expression response in fatigue-loaded rat patellar tendons. J. Orthop. Res..

[35] Zhang J., Pan T., Liu Y., Wang J.H.-C. (2010). Mouse treadmill running enhances tendons by expanding the pool of tendon stem cells (TSCs) and TSC-related cellular production of collagen. J. Orthop. Res..

[36] Szczodry M., Zhang J., Lim C., Davitt H.L., Yeager T., Fu F.H., Wang J.H.-C. (2009). Treadmill running exercise results in the presence of numerous myofibroblasts in mouse patellar tendons. J. Orthop. Res..

[37] Woo S.L.-Y. (2009). Tissue engineering: Use of scaffolds for ligament and tendon healing and regeneration. Knee Surg. Sports Traumatol. Arthrosc..

[38] Sharma P., Maffulli N. (2006). Biology of tendon injury: Healing, modeling and remodeling. J. Musculoskelet. Neuronal Interact..

[39] Woo S.L.-Y., Hildebrand K., Watanabe N., Fenwick J.A., Papageorgiou C.D., Wang J.H.-C. (1999). Tissue engineering of ligament and tendon healing. Clin. Orthop. Relat. Res..

[40] Woo S.L.-Y., Buckwalter J.A. (1988). Injury and repair of the musculoskeletal soft tissues. J. Orthop. Res..

[41] Oshiro W., Lou J., Xing X., Tu Y., Manske P.R. (2003). Flexor tendon healing in the rat: A histologic and gene expression study. J. Hand Surg. Am..

[42] Nichols A.E.C., Best K.T., Loiselle A.E. (2019). The cellular basis of fibrotic tendon healing: Challenges and opportunities. Transl. Res..

[43] Baldino L., Cardea S., Maffulli N., Reverchon E. (2016). Regeneration techniques for bone-to-tendon and muscle-to-tendon interfaces reconstruction. Br. Med. Bull..

[44] Yamamoto A., Takagishi K., Osawa T., Yanagawa T., Nakajima D., Shitara H., Kobayashi T. (2010). Prevalence and risk factors of a rotator cuff tear in the general population. J. Shoulder Elb. Surg..

[45] Galvin J.W., Waterman B.R., Cole B.J. (2018). Patch augmentation and patch extension for complex rotator cuff tears. Oper. Tech. Sports Med..

[46] Maitz M.F. (2015). Applications of synthetic polymers in clinical medicine. Biosurf. Biotribol..

[47] Ruiz-Alonso S., Lafuente-Merchan M., Ciriza J., Saenz-del-Burgo L., Pedraz J.L. (2021). Tendon tissue engineering: Cells, growth factors, scaffolds and production techniques. J. Control. Release.

[48] Shiroud Heidari B., Ruan R., Vahabli E., Chen P., De-Juan-Pardo E.M., Zheng M., Doyle B. (2023). Natural, synthetic and commercially-available biopolymers used to regenerate tendons and ligaments. Bioact. Mater..

[49] Woodard L.N., Grunlan M.A. (2018). Hydrolytic degradation and erosion of polyester biomaterials. ACS Macro Lett..

[50] Gorgieva S., Kokol V., Pignatello R. (2011). Collagen-vs. gelatine-based biomaterials and their biocompatibility: Review and perspectives. Biomaterials Applications for Nanomedicine.

[51] Subramanian G., Stasuk A., Elsaadany M., Yildirim-Ayan E. (2017). Effect of uniaxial tensile cyclic loading regimes on matrix organization and tenogenic differentiation of adipose-derived stem cells encapsulated within 3D collagen scaffolds. Stem Cells Int..

[52] Thomopoulos S., Das R., Birman V., Smith L., Ku K., Elson E.L., Pryse K.M., Marquez J.P., Genin G.M. (2011). Fibrocartilage tissue engineering: The role of the stress environment on cell morphology and matrix expression. Tissue Eng. Part A.

[53] Yang G., Lin H., Rothrauff B.B., Yu S., Tuan R.S. (2016). Multilayered polycaprolactone/gelatin fiber-hydrogel composite for tendon tissue engineering. Acta Biomater..

[54] Li H., Fan J., Sun L., Liu X., Cheng P., Fan H. (2016). Functional regeneration of ligament-bone interface using a triphasic silk-based graft. Biomaterials.

[55] Silva M., Ferreira F.N., Alves N.M., Paiva M.C. (2020). Biodegradable polymer nanocomposites for ligament/tendon tissue engineering. J. Nanobiotechnol..

[56] Sensini A., Gualandi C., Cristofolini L., Tozzi G., Dicarlo M., Teti G., Mattioli-Belmonte M., Focarete M.L. (2017). Biofabrication of bundles of poly(lactic acid)-collagen blends mimicking the fascicles of the human Achille tendon. Biofabrication.

[57] Calejo I., Costa-Almeida R., Reis R.L., Gomes M.E. (2019). A textile platform using continuous aligned and textured composite microfibers to engineer tendon-to-bone interface gradient scaffolds. Adv. Healthc. Mater..

[58] Weinreb J.H., Sheth C., Apostolakos J., McCarthy M.B., Barden B., Cote M.P., Mazzocca A.D. (2014). Tendon structure, disease, and imaging. Muscles Ligaments Tendons J..

[59] Cardwell R.D., Dahlgren L.A., Goldstein A.S. (2014). Electrospun fibre diameter, not alignment, affects mesenchymal stem cell differentiation into the tendon/ligament lineage. J. Tissue Eng. Regen. Med..

[60] Bashur C.A., Shaffer R.D., Dahlgren L.A., Guelcher S.A., Goldstein A.S. (2009). Effect of fiber diameter and alignment of electrospun polyurethane meshes on mesenchymal progenitor cells. Tissue Eng. Part A.

[61] Erisken C., Zhang X., Moffat K.L., Levine W.N., Lu H.H. (2013). Scaffold fiber diameter regulates human tendon fibroblast growth and differentiation. Tissue Eng. Part A.

[62] Sensini A., Cristofolini L. (2018). Biofabrication of electrospun scaffolds for the regeneration of tendons and ligaments. Materials.

[63] Caliari S.R., Harley B.A.C. (2011). The effect of anisotropic collagen-GAG scaffolds and growth factor supplementation on tendon cell recruitment, alignment, and metabolic activity. Biomaterials.

[64] Whitlock P.W., Smith T.L., Poehling G.G., Shilt J.S., Van Dyke M. (2007). A naturally derived, cytocompatible, and architecturally optimized scaffold for tendon and ligament regeneration. Biomaterials.

[65] Zheng Z., Ran J., Chen W., Hu Y., Zhu T., Chen X., Yin Z., Heng B.C., Feng G., Le H. (2017). Alignment of collagen fiber in knitted silk scaffold for functional massive rotator cuff repair. Acta Biomater..

[66] Schoenenberger A.D., Tempfer H., Lehner C., Egloff J., Mauracher M., Bird A., Widmer J., Maniura-Weber K., Fucentese S.F., Traweger A. (2020). Macromechanics and polycaprolactone fiber organization drive macrophage polarization and regulate inflammatory activation of tendon in vitro and in vivo. Biomaterials.

[67] Schoenenberger A.D., Foolen J., Moor P., Silvan U., Snedeker J.G. (2018). Substrate fiber alignment mediates tendon cell response to inflammatory signaling. Acta Biomater..

[68] Orr S.B., Chainani A., Hippensteel K.J., Kishan A., Gilchrist C., Garrigues N.W., Ruch D.S., Guilak F., Little D. (2015). Aligned multilayered electrospun scaffolds for rotator cuff tendon tissue engineering. Acta Biomater..

[69] Dai Y., Li X., Wu R., Jin Y., Gao C. (2018). Macrophages of different phenotypes influence the migration of BMSCs in PLGA scaffolds with different pore size. Biotechnol. J..

[70] Saino E., Focarete M.L., Gualandi C., Emanuele E., Cornaglia A.I., Imbriani M., Visai L. (2011). Effect of electrospun fiber diameter and alignment on macrophage activation and secretion of proinflammatory cytokines and chemokines. Biomacromolecules.

[71] Grier W.K., Iyoha E.M., Harley B.A.C. (2017). The influence of pore size and stiffness on tenocyte bioactivity and transcriptomic stability in collagen-GAG scaffolds. J. Mech. Behav. Biomed. Mater..

[72] Sridharan R., Cavanagh B., Cameron A.R., Kelly D.J., O’Brien F.J. (2019). Material stiffness influences the polarization state, function and migration mode of macrophages. Acta Biomater..

[73] Previtera M.L., Sengupta A. (2015). Substrate stiffness regulates proinflammatory mediator production through TLR4 activity in macrophages. PLoS ONE.

[74] Blakney A.K., Swartzlander M.D., Bryant S.J. (2012). The effects of substrate stiffness on the in vitro activation of macrophages and in vivo host response to poly(ethylene glycol)-based hydrogels. J. Biomed. Mater. Res. A.

[75] Liu C., Luo J.-W., Liang T., Lin L.-X., Luo Z.-P., Zhuang Y.-Q., Sun Y.-L. (2018). Matrix stiffness regulates the differentiation of tendon-derived stem cells through FAK-ERK1/2 activation. Exp. Cell Res..

[76] Liu C., Tian S., Bai J., Yu K., Liu L., Liu G., Dong R., Tian D. (2020). Regulation of ERK1/2 and SMAD2/3 pathways by using multi-layered electrospun PCL–amnion nanofibrous membranes for the prevention of post-surgical tendon adhesion. Int. J. Nanomed..

[77] Echeverria Molina M.I., Malollari K.G., Komvopoulos K. (2021). Design challenges in polymeric scaffolds for tissue engineering. Front. Bioeng. Biotechnol..

[78] Pucino V., Certo M., Bulusu V., Cucchi D., Goldmann K., Pontarini E., Haas R., Smith J., Headland S.E., Blighe K. (2019). Lactate buildup at the site of chronic inflammation promotes disease by inducing CD4+ T cell metabolic rewiring. Cell Metab..

[79] Holloway J.L., Ma H., Rai R., Burdick J.A. (2014). Modulating hydrogel crosslink density and degradation to control bone morphogenetic protein delivery and in vivo bone formation. J. Control. Release.

[80] Brannigan R.P., Dove A.P. (2017). Synthesis, properties and biomedical applications of hydrolytically degradable materials based on aliphatic polyesters and polycarbonates. Biomater. Sci..

[81] Kianpour G., Bagheri R., Pourjavadi A., Ghanbari H. (2020). In situ synthesized TiO_2_-polyurethane nanocomposite for bypass graft application: In vitro endothelialization and degradation. Mater. Sci. Eng. C.

[82] Annor A.H., Tang M.E., Pui C.L., Ebersole G.C., Frisella M.M., Matthews B.D., Deeken C.R. (2012). Effect of enzymatic degradation on the mechanical properties of biological scaffold materials. Surg. Endosc..

[83] Fu W., Liu Z., Feng B., Hu R., He X., Wang H., Yin M., Huang H., Zhang H., Wang W. (2014). Electrospun gelatin/PCL and collagen/PLCL scaffolds for vascular tissue engineering. Int. J. Nanomed..

[84] Spagnuolo M., Liu L. (2012). Fabrication and degradation of electrospun scaffolds from L-tyrosine-based polyurethane blends for tissue engineering applications. ISRN Nanotechnol..

[85] Ma C.H., Zhang H.B., Yang S.M., Yin R.X., Yao X.J., Zhang W.J. (2018). Comparison of the degradation behavior of PLGA scaffolds in micro-channel, shaking, and static conditions. Biomicrofluidics.

[86] Rediguieri C.F., Sassonia R.C., Dua K., Kikuchi I.S., Pinto T.J.A. (2016). Impact of sterilization methods on electrospun scaffolds for tissue engineering. Eur. Polym. J..

[87] Cooke S.L., Whittington A.R. (2016). Influence of therapeutic radiation on polycaprolactone and polyurethane biomaterials. Mater. Sci. Eng. C.

[88] Jones E.M., Cochrane C.A., Percival S.L. (2015). The effect of pH on the extracellular matrix and biofilms. Adv. Wound Care.

[89] Sharpe J.R., Harris K.L., Jubin K., Bainbridge N.J., Jordan N.R. (2009). The effect of pH in modulating skin cell behaviour. Br. J. Dermatol..

[90] Dellacherie M.O., Seo B.R., Mooney D.J. (2019). Macroscale biomaterials strategies for local immunomodulation. Nat. Rev. Mater..

[91] Stabler C.L., Li Y., Stewart J.M., Keselowsky B.G. (2019). Engineering immunomodulatory biomaterials for type 1 diabetes. Nat. Rev. Mater..

[92] Dziki J.L., Badylak S.F. (2018). Immunomodulatory biomaterials. Curr. Opin. Biomed. Eng..

[93] Sussman E.M., Halpin M.C., Muster J., Moon R.T., Ratner B.D. (2014). Porous implants modulate healing and induce shifts in local macrophage polarization in the foreign body reaction. Ann. Biomed. Eng..

[94] Ye Q., Harmsen M.C., van Luyn M.J.A., Bank R.A. (2010). The relationship between collagen scaffold cross-linking agents and neutrophils in the foreign body reaction. Biomaterials.

[95] Sadtler K., Estrellas K., Allen B.W., Wolf M.T., Fan H., Tam A.J., Patel C.H., Luber B.S., Wang H., Wagner K.R. (2016). Developing a pro-regenerative biomaterial scaffold microenvironment requires T helper 2 cells. Science.

[96] Brown B.N., Londono R., Tottey S., Zhang L., Kukla K.A., Wolf M.T., Daly K.A., Reing J.E., Badylak S.F. (2012). Macrophage phenotype as a predictor of constructive remodeling following the implantation of biologically derived surgical mesh materials. Acta Biomater..

[97] Veiseh O., Doloff J.C., Ma M., Vegas A.J., Tam H.H., Bader A.R., Li J., Langan E., Wyckoff J., Loo W.S. (2015). Size- and shape-dependent foreign body immune response to materials implanted in rodents and non-human primates. Nat. Mater..

[98] Sadtler K., Sommerfeld S.D., Wolf M.T., Wang X., Majumdar S., Chung L., Kelkar D.S., Pandey A., Elisseeff J.H. (2017). Proteomic composition and immunomodulatory properties of urinary bladder matrix scaffolds in homeostasis and injury. Semin. Immunol..

[99] Anderson J.M., Rodriguez A., Chang D.T. (2008). Foreign body reaction to biomaterials. Semin. Immunol..

[100] Celikkin N., Rinoldi C., Costantini M., Trombetta M., Rainer A., Święszkowski W. (2017). Naturally derived proteins and glycosaminoglycan scaffolds for tissue engineering applications. Mater. Sci. Eng. C.

[101] Wu Y., Han Y., Wong Y.S., Fuh J.Y.H. (2018). Fibre-based scaffolding techniques for tendon tissue engineering. J. Tissue Eng. Regen. Med..

[102] Chainani A., Little D. (2016). Current status of tissue-engineered scaffolds for rotator cuff repair. Tech. Orthop..

[103] Idaszek J., Kijeńska E., Łojkowski M., Święszkowski W. (2016). How important are scaffolds and their surface properties in regenerative medicine. Appl. Surf. Sci..

[104] Lee J., Guarino V., Gloria A., Ambrosio L., Tae G., Kim Y.H., Jung Y., Kim S.H., Kim S.H. (2010). Regeneration of Achilles’ tendon: The role of dynamic stimulation for enhanced cell proliferation and mechanical properties. J. Biomater. Sci. Polym. Ed..

[105] Fang F., Lake S.P. (2017). Experimental evaluation of multiscale tendon mechanics. J. Orthop. Res..

[106] Pauly H.M., Kelly D.J., Popat K.C., Trujillo N.A., Dunne N.J., McCarthy H.O., Haut Donahue T.L. (2016). Mechanical properties and cellular response of novel electrospun nanofibers for ligament tissue engineering: Effects of orientation and geometry. J. Mech. Behav. Biomed. Mater..

[107] Nau T., Teuschl A. (2015). Regeneration of the anterior cruciate ligament: Current strategies in tissue engineering. World J. Orthop..

[108] Kumar N., Sharma A.K., Singh G.R., Gupta O.P. (2002). Carbon fibres and plasma-preserved tendon allografts for gap repair of flexor tendon in bovines: Clinical, radiological and angiographical observations. J. Veter. Med. Ser. A.

[109] Panas-Perez E., Gatt C.J., Dunn M.G. (2013). Development of a silk and collagen fiber scaffold for anterior cruciate ligament reconstruction. J. Mater. Sci. Mater. Med..

[110] Rinoldi C., Kijeńska-Gawrońska E., Khademhosseini A., Tamayol A., Swieszkowski W. (2018). Nanobead-on-string composites for tendon tissue engineering. J. Mater. Chem. B.

[111] Tu T., Shen Y., Wang X., Zhang W., Zhou G., Zhang Y., Wang W., Liu W. (2020). Tendon ECM modified bioactive electrospun fibers promote MSC tenogenic differentiation and tendon regeneration. Appl. Mater. Today.

[112] Maghdouri-White Y., Bowlin G.L., Lemmon C.A., Dréau D. (2018). Electrospun silk-collagen scaffolds and BMP-13 for ligament and tendon repair and regeneration. Biomed. Phys. Eng. Express.

[113] Leciejewski M., Królikowska A., Reichert P. (2018). Polyethylene terephthalate tape augmentation as a solution in recurrent quadriceps tendon ruptures. Polym. Med..

[114] Longo U.G., Lamberti A., Maffulli N., Denaro V. (2010). Tendon augmentation grafts: A systematic review. Br. Med. Bull..

[115] Learn G.D., McClellan P.E., Knapik D.M., Cumsky J.L., Webster-Wood V., Anderson J.M., Gillespie R.J., Akkus O. (2019). Woven collagen biotextiles enable mechanically functional rotator cuff tendon regeneration during repair of segmental tendon defects in vivo. J. Biomed. Mater. Res. B Appl. Biomater..

[116] Ruan D., Zhu T., Huang J., Le H., Hu Y., Zheng Z., Tang C., Chen Y., Ran J., Chen X. (2019). Knitted silk-collagen scaffold incorporated with ligament stem/progenitor cells sheet for anterior cruciate ligament reconstruction and osteoarthritis prevention. ACS Biomater. Sci. Eng..

[117] Cristino S., Grassi F., Toneguzzi S., Piacentini A., Grigolo B., Santi S., Riccio M., Tognana E., Facchini A., Lisignoli G. (2005). Analysis of mesenchymal stem cells grown on a three-dimensional HYAFF 11^®^-based prototype ligament scaffold. J. Biomed. Mater. Res. A.

[118] Vaquette C., Kahn C., Frochot C., Nouvel C., Six J.-L., De Isla N., Luo L.-H., Cooper-White J., Rahouadj R., Wang X. (2010). Aligned poly(L-lactic-co-ε-caprolactone) electrospun microfibers and knitted structure: A novel composite scaffold for ligament tissue engineering. J. Biomed. Mater. Res. A.

[119] Czaplewski S.K., Tsai T.L., Duenwald-Kuehl S.E., Vanderby R., Li W.J. (2014). Tenogenic differentiation of human induced pluripotent stem cell-derived mesenchymal stem cells dictated by properties of braided submicron fibrous scaffolds. Biomaterials.

[120] Kumar K., Maffulli N. (1999). The ligament augmentation device: An historical perspective. Arthroscopy.

[121] Gwiazda M., Kumar S., Świeszkowski W., Ivanovski S., Vaquette C. (2020). The effect of melt electrospun writing fiber orientation onto cellular organization and mechanical properties for application in anterior cruciate ligament tissue engineering. J. Mech. Behav. Biomed. Mater..

[122] Sandri M., Filardo G., Kon E., Panseri S., Montesi M., Iafisco M., Savini E., Sprio S., Cunha C., Giavaresi G. (2016). Fabrication and pilot in vivo study of a collagen-BDDGE-elastin core-shell scaffold for tendon regeneration. Front. Bioeng. Biotechnol..

[123] Freeman J.W., Woods M.D., Laurencin C.T. (2007). Tissue engineering of the anterior cruciate ligament using a braid-twist scaffold design. J. Biomech..

[124] Freeman J.W., Woods M.D., Cromer D.A., Wright L.D., Laurencin C.T. (2009). Tissue engineering of the anterior cruciate ligament: The viscoelastic behavior and cell viability of a novel braid-twist scaffold. J. Biomater. Sci. Polym. Ed..

[125] Rinoldi C., Kijeńska-Gawrońska E., Khademhosseini A., Tamayol A., Swieszkowski W. (2021). Fibrous systems as potential solutions for tendon and ligament repair, healing, and regeneration. Adv. Healthc. Mater..

[126] Rashid M., Dudhia J., Dakin S.G., Snelling S.J.B., De Godoy R., Mouthuy P.-A., Smith R.K.W., Morrey M., Carr A.J. (2020). Histopathological and immunohistochemical evaluation of cellular response to a woven and electrospun polydioxanone (PDO) and polycaprolactone (PCL) patch for tendon repair. Sci. Rep..

[127] Sharifi-Aghdam M., Faridi-Majidi R., Derakhshan M.A., Chegeni A., Azami M. (2017). Preparation of collagen/polyurethane/knitted silk as a composite scaffold for tendon tissue engineering. Proc. Inst. Mech. Eng. H.

[128] Romeo A., Easley J., Regan D., Hackett E., Johnson J., Johnson J., Puttlitz C., McGilvray K. (2022). Rotator cuff repair using a bioresorbable nanofiber interposition scaffold: A biomechanical and histologic analysis in sheep. J. Shoulder Elb. Surg..

[129] Silva M., Gomes S., Correia C., Peixoto D., Vinhas A., Rodrigues M.T., Gomes M.E., Covas J.A., Paiva M.C., Alves N.M. (2023). Biocompatible 3D-printed tendon/ligament scaffolds based on polylactic acid/graphite nanoplatelet composites. Nanomaterials.

[130] Yao K., Lv S., Zhang X., Shen K., Chen Y., Ma Z., He Y. (2025). 3D printing of multiscale biomimetic scaffold for tendon regeneration. Adv. Funct. Mater..

[131] Chen Y.C., Gad S.F., Chobisa D., Li Y., Yeo Y. (2021). Local drug delivery systems for inflammatory diseases: Status quo, challenges, and opportunities. J. Control. Release.

[132] Campbell S., Smeets N., Kobayashi S., Müllen K. (2019). Drug delivery: Localized and systemic therapeutic strategies with polymer systems. Encyclopedia of Polymeric Nanomaterials.

[133] Jacob S., Nair A.B., Shah J., Sreeharsha N., Gupta S., Shinu P. (2021). Emerging role of hydrogels in drug delivery systems, tissue engineering and wound management. Pharmaceutics.

[134] Adjei-Sowah E., Benoit D.S.W., Loiselle A.E. (2023). Drug delivery approaches to improve tendon healing. Tissue Eng. Part B Rev..

[135] Pasternak B., Missios A., Askendal A., Tengvall P., Aspenberg P. (2007). Doxycycline-coated sutures improve the suture-holding capacity of the rat Achilles tendon. Acta Orthop..

[136] Zhou Y.L., Yang Q.Q., Yan Y.Y., Zhu C., Zhang L., Tang J.B. (2019). Gene-loaded nanoparticle-coated sutures provide effective gene delivery to enhance tendon healing. Mol. Ther..

[137] Garg T., Singh O., Arora S., Murthy R.S.R. (2012). Scaffold: A novel carrier for cell and drug delivery. Crit. Rev. Ther. Drug Carr. Syst..

[138] Lei T., Zhang T., Ju W., Chen X., Heng B.C., Shen W., Yin Z. (2021). Biomimetic strategies for tendon/ligament-to-bone interface regeneration. Bioact. Mater..

[139] Stratton S., Shelke N.B., Hoshino K., Rudraiah S., Kumbar S.G. (2016). Bioactive polymeric scaffolds for tissue engineering. Bioact. Mater..

[140] Hu C., Liu S., Zhang Y., Li B., Yang H., Fan C., Cui W. (2013). Long-term drug release from electrospun fibers for in vivo inflammation prevention in the prevention of peritendinous adhesions. Acta Biomater..

[141] Chen E., Yang L., Ye C., Zhang W., Ran J., Xue D., Wang Z., Pan Z., Hu Q. (2018). An asymmetric chitosan scaffold for tendon tissue engineering: In vitro and in vivo evaluation with rat tendon stem/progenitor cells. Acta Biomater..

[142] Parchi P.D., Vittorio O., Andreani L., Piolanti N., Cirillo G., Di Iorio J., Marchetti S., Poggetti A., Lisanti M. (2016). Nanoparticles for tendon healing and regeneration: Literature review. Front. Aging Neurosci..

[143] Fan Y., Marioli M., Zhang K. (2021). Analytical characterization of liposomes and other lipid nanoparticles for drug delivery. J. Pharm. Biomed. Anal..

[144] Chen Y., Shen W., Tang C., Huang J., Fan C., Yin Z., Hu Y., Chen W., Ouyang H., Zhou Y. (2020). Targeted pathological collagen delivery of sustained-release rapamycin to prevent heterotopic ossification. Sci. Adv..

[145] Sims K.R., Maceren J.P., Liu Y., Rocha G.R., Koo H., Benoit D.S.W. (2020). Dual antibacterial drug-loaded nanoparticles synergistically improve treatment of *Streptococcus mutans* biofilms. Acta Biomater..

[146] Cheng R., Meng F., Deng C., Klok H.-A., Zhong Z. (2013). Dual and multi-stimuli responsive polymeric nanoparticles for programmed site-specific drug delivery. Biomaterials.

[147] Zeng Y., Zhang X., Zeng Y., Zhang X., Yu Z., Zhang Y., Wang H., Li M., Wang J., Zhang X. (2018). Hypoxia-activated prodrugs and redox-responsive nanocarriers. Int. J. Nanomed..

[148] Ponnapakkam T., Katikaneni R., Sakon J., Stratford R., Gensure R.C. (2011). Monthly administration of a novel PTH-collagen binding domain fusion protein is anabolic in mice. Calcif. Tissue Int..

[149] Ponnapakkam T., Katikaneni R., Sakon J., Stratford R., Gensure R.C. (2014). Treating osteoporosis by targeting parathyroid hormone to bone. Drug Discov. Today.

[150] Ponnapakkam T., Katikaneni R., Sakon J., Stratford R., Gensure R.C. (2012). A single injection of the anabolic bone agent, parathyroid hormone-collagen binding domain (PTH-CBD), results in sustained increases in bone mineral density for up to 12 months in normal female mice. Calcif. Tissue Int..

[151] Chen Z., Jin M., He H., Dong J., Li J., Nie J., Wang Z., Xu J., Wu F. (2023). Mesenchymal stem cells and macrophages and their interactions in tendon-bone healing. J. Orthop. Transl..

[152] Malekpour K., Hazrati A., Zahar M., Markov A., Zekiy A.O., Navashenaq J.G., Roshangar L., Ahmadi M. (2021). The potential use of mesenchymal stem cells and their derived exosomes for orthopedic diseases treatment. Stem Cell Rev. Rep..

[153] Xu Y., Zhang W.X., Wang L.N., Ming Y.Q., Li Y.L., Ni G.X. (2021). Stem cell therapies in tendon-bone healing. World J. Stem Cells.

[154] Fu X., Liu G., Halim A., Ju Y., Luo Q., Song G. (2019). Mesenchymal stem cell migration and tissue repair. Cells.

[155] Torres-Torrillas M., Rubio M., Damia E., Cuervo B., del Romero A., Peláez P., Chicharro D., Miguel L., Sopena J.J. (2019). Adipose-derived mesenchymal stem cells: A promising tool in the treatment of musculoskeletal diseases. Int. J. Mol. Sci..

[156] Jiang L., Lu J., Chen Y., Lyu K., Long L., Wang X., Liu T., Li S. (2023). Mesenchymal stem cells: An efficient cell therapy for tendon repair (Review). Int. J. Mol. Med..

[157] Migliorini F., Tingart M., Maffulli N. (2020). Progress with stem cell therapies for tendon tissue regeneration. Expert Opin. Biol. Ther..

[158] Yea J.H., Kim Y., Jo C.H. (2023). Comparison of mesenchymal stem cells from bone marrow, umbilical cord blood, and umbilical cord tissue in regeneration of a full-thickness tendon defect in vitro and in vivo. Biochem. Biophys. Rep..

[159] Baberg F., Geyh S., Waldera-Lupa D., Stefanski A., Zilkens C., Haas R., Schroeder T., Stühler K. (2019). Secretome analysis of human bone marrow derived mesenchymal stromal cells. Biochim. Biophys. Acta Proteins Proteom..

[160] Yea J.H., Bae T.S., Kim B.J., Cho Y.W., Jo C.H. (2020). Regeneration of the rotator cuff tendon-to-bone interface using umbilical cord-derived mesenchymal stem cells and gradient extracellular matrix scaffolds from adipose tissue in a rat model. Acta Biomater..

[161] Lim W.L., Chowdhury S.R., Ng M.H., Law J.X. (2021). Physicochemical properties and biocompatibility of electrospun polycaprolactone/gelatin nanofibers. Int. J. Environ. Res. Public Health.

[162] Ciardulli M.C., Marino L., Lovecchio J., Giordano E., Forsyth N.R., Selleri C., Maffulli N., Della Porta G. (2020). Tendon and cytokine marker expression by human bone marrow mesenchymal stem cells in a hyaluronate/poly-lactic-co-glycolic acid (PLGA)/fibrin three-dimensional (3D) scaffold. Cells.

[163] Smith R.K.W., Werling N.J., Dakin S.G., Alam R., Goodship A.E., Dudhia J. (2013). Beneficial effects of autologous bone marrow-derived mesenchymal stem cells in naturally occurring tendinopathy. PLoS ONE.

[164] Lee C.H., Cook J.L., Mendelson A., Moioli E.K., Yao H., Mao J.J. (2010). Regeneration of the articular surface of the rabbit synovial joint by cell homing: A proof of concept study. Lancet.

[165] Shi Y., Kang X., Wang Y., Bian X., He G., Zhou M., Tang K. (2020). Exosomes derived from bone marrow stromal cells (BMSCs) enhance tendon-bone healing by regulating macrophage polarization. Med. Sci. Monit..

[166] Heo J.S., Choi Y., Kim H.O., Matta C. (2019). Adipose-derived mesenchymal stem cells promote M2 macrophage phenotype through exosomes. Stem Cells Int..

[167] Fu G., Lu L., Pan Z., Fan A., Yin F. (2021). Adipose-derived stem cell exosomes facilitate rotator cuff repair by mediating tendon-derived stem cells. Regen. Med..

[168] Liu H., Zhang M., Shi M., Zhang T., Lu W., Yang S., Cui Q., Li Z. (2021). Adipose-derived mesenchymal stromal cell-derived exosomes promote tendon healing by activating both SMAD1/5/9 and SMAD2/3. Stem Cell Res. Ther..

[169] Wu X.D., Kang L., Tian J., Wu Y., Huang Y., Liu J., Wang H., Qiu G., Wu Z. (2022). Exosomes derived from magnetically actuated bone mesenchymal stem cells promote tendon-bone healing through the miR-21-5p/SMAD7 pathway. Mater. Today Bio.

[170] Arvind V., Huang A.H. (2021). Reparative and maladaptive inflammation in tendon healing. Front. Bioeng. Biotechnol..

[171] Shi Y., Wang Y., Li Q., Liu K., Hou J., Shao C., Wang Y. (2018). Immunoregulatory mechanisms of mesenchymal stem and stromal cells in inflammatory diseases. Nat. Rev. Nephrol..

[172] Kokubu S., Inaki R., Hoshi K., Hikita A. (2020). Adipose-derived stem cells improve tendon repair and prevent ectopic ossification in tendinopathy by inhibiting inflammation and inducing neovascularization in the early stage of tendon healing. Regen. Ther..

[173] Huang Y., He B., Wang L., Yuan B., Shu H., Zhang F., Sun L. (2020). Bone marrow mesenchymal stem cell-derived exosomes promote rotator cuff tendon-bone healing by promoting angiogenesis and regulating M1 macrophages in rats. Stem Cell Res. Ther..

[174] Li Z., Li Q., Tong K., Zhu J., Wang H., Chen B., Chen L. (2022). BMSC-derived exosomes promote tendon-bone healing after anterior cruciate ligament reconstruction by regulating M1/M2 macrophage polarization in rats. Stem Cell Res. Ther..

[175] Taylor C.T. (2008). Interdependent roles for hypoxia inducible factor and nuclear factor-κB in hypoxic inflammation. J. Physiol..

[176] Berse B., A Hunt J., Diegel R.J., Morganelli P., Yeo K.T., Brown F., Fava R.A. (1999). Hypoxia augments cytokine (transforming growth factor-beta (TGF-β) and IL-1)-induced vascular endothelial growth factor secretion by human synovial fibroblasts. Clin. Exp. Immunol..

[177] Kaigler D., Krebsbach P.H., Polverini P.J., Mooney D.J. (2003). Role of vascular endothelial growth factor in bone marrow stromal cell modulation of endothelial cells. Tissue Eng..

[178] Furumatsu T., Shen Z.N., Kawai A., Nishida K., Manabe H., Oohashi T., Inoue H., Ninomiya Y. (2003). Vascular endothelial growth factor principally acts as the main angiogenic factor in the early stage of human osteoblastogenesis. J. Biochem..

[179] Al-Khaldi A., Eliopoulos N., Martineau D., Lejeune L., Lachapelle K., Galipeau J. (2003). Postnatal bone marrow stromal cells elicit a potent VEGF-dependent neoangiogenic response in vivo. Gene Ther..

[180] Qu Q., Wang L., Bing W., Bi Y., Zhang C., Jing X., Liu L. (2020). miRNA-126-3p carried by human umbilical cord mesenchymal stem cell enhances endothelial function through exosome-mediated mechanisms in vitro and attenuates vein graft neointimal formation in vivo. Stem Cell Res. Ther..

[181] Gong M., Yu B., Wang J., Wang Y., Liu M., Paul C., Millard R.W., Xiao D.-S., Ashraf M., Xu M. (2017). Mesenchymal stem cells release exosomes that transfer miRNAs to endothelial cells and promote angiogenesis. Oncotarget.

[182] Voleti P.B., Buckley M.R., Soslowsky L.J. (2012). Tendon healing: Repair and regeneration. Annu. Rev. Biomed. Eng..

[183] Qin S., Wang W., Liu Z., Hua X., Fu S., Dong F., Li A., Liu Z., Wang P., Dai L. (2020). Fibrochondrogenic differentiation potential of tendon-derived stem/progenitor cells from human patellar tendon. J. Orthop. Transl..

[184] Leong D.J., Sun H.B. (2016). Mesenchymal stem cells in tendon repair and regeneration: Basic understanding and translational challenges. Ann. N. Y. Acad. Sci..

[185] Steinmann S., Pfeifer C.G., Brochhausen C., Docheva D. (2020). Spectrum of tendon pathologies: Triggers, trails and end-state. Int. J. Mol. Sci..

[186] Hoeffner R., Svensson R.B., Bjerregaard N., Kjær M., Magnusson S.P. (2022). Persistent deficits after an Achilles tendon rupture: A narrative review. Transl. Sports Med..

[187] Rawson S., Cartmell S., Wong J. (2013). Suture techniques for tendon repair: A comparative review. Muscles Ligaments Tendons J..

[188] Jennings M.M., Liew V., Marine B. (2019). Updates in tendinopathy treatment options. Clin. Podiatr. Med. Surg..

[189] Pluchino M., Vivarelli L., Giavaresi G., Dallari D., Govoni M. (2025). Commercial biomaterial-based products for tendon surgical augmentation: A scoping review on currently available medical devices. J. Funct. Biomater..

[190] Flury M., Rickenbacher D., Jung C., Schneider M.M., Endell D., Audigé L. (2018). Porcine dermis patch augmentation of supraspinatus tendon repairs: A pilot study assessing tendon integrity and shoulder function 2 years after arthroscopic repair in patients aged 60 years or older. Arthroscopy.

[191] Ruiz Ibán M.Á., Navlet M.G., Marco S.M., Heredia J.D., Sánchez A.H., Díaz R.R., Comino C.V., Ojeda M.L.R., Bello G.d.M., Lafuente J.L.Á. (2024). Augmentation of a transosseous-equivalent repair in posterosuperior nonacute rotator cuff tears with a bioinductive collagen implant decreases the retear rate at 1 year: A randomized controlled trial. Arthroscopy.

[192] Schlegel T.F., Abrams J.S., Angelo R.L., Getelman M.H., Ho C.P., Bushnell B.D. (2021). Isolated bioinductive repair of partial-thickness rotator cuff tears using a resorbable bovine collagen implant: Two-year radiologic and clinical outcomes from a prospective multicenter study. J. Shoulder Elb. Surg..

[193] Thakker A., Sharma S.C., Hussain N.M., Devani P., Lahiri A. (2021). Nerve wrapping for recurrent compression neuropathy: A systematic review. J. Plast. Reconstr. Aesthet. Surg..

[194] Hones K.M., Nichols D.S., Barker H., Cox E., Hones J.A., Chim H. (2023). Outcomes following use of VersaWrap nerve protector in treatment of patients with recurrent compressive neuropathies. Front. Surg..

[195] Adu Y., Harder J., Cox C., Baum G., Hernandez E.J., MacKay B.J. (2024). Evaluating the effect of VersaWrap tendon protector on functional outcomes in operative tendon repairs. Front. Surg..

[196] Proctor C.S. (2014). Long-term successful arthroscopic repair of large and massive rotator cuff tears with a functional and degradable reinforcement device. J. Shoulder Elb. Surg..

[197] Barbash S., Denny C., Collin P., Reish T., Hart J.M., Brockmeier S.F. (2015). Clinical outcomes and structural healing after arthroscopic rotator cuff repair reinforced with a novel absorbable biologic scaffold: A prospective, multicenter trial. Orthop. J. Sports Med..

[198] Smolen D., Haffner N., Mittermayr R., Hess F., Sternberg C., Leuzinger J. (2020). Application of a new polyester patch in arthroscopic massive rotator cuff repair—A prospective cohort study. J. Shoulder Elb. Surg..

[199] Solan C., Starring H., Reese J., Leithead C., Galli S. (2023). Simultaneous extensor hallicus longus tendon laceration and dorsalis pedis artery pseudoaneurysm: A case report and a review of the literature. Foot Ankle Surg. Tech. Rep. Cases.

[200] Alencar Mendes de Carvalho K., Schmidt E., Ehret A., Chun Kim K., Barbachan Mansur N.S., De César Netto C. (2022). Achilles tendon reconstruction using a biosynthetic graft: A case report. J. Foot Ankle.

[201] Walsh W.R., Batista J., Lovric V., Crowley J.D., Wills D., Carter A.J., Wang T., Stanton R., Rocco K., Arciero R. (2026). Augmentation of a rotator cuff tendon repair using a bioinductive biocomposite scaffold in an ovine infraspinatus model. J. Shoulder Elb. Surg..

